# Long-read metagenomics gives a more accurate insight into the microbiota of long-ripened gouda cheeses

**DOI:** 10.3389/fmicb.2025.1543079

**Published:** 2025-03-24

**Authors:** Hannes Decadt, Cristian Díaz-Muñoz, Louise Vermote, Inés Pradal, Luc De Vuyst, Stefan Weckx

**Affiliations:** Research Group of Industrial Microbiology and Food Biotechnology (IMDO), Faculty of Sciences and Bioengineering Sciences, Vrije Universiteit Brussel, Brussels, Belgium

**Keywords:** Illumina short-read sequencing, Oxford Nanopore Technologies long-read sequencing, metagenome-assembled genomes, intraspecies metagenomics, high-throughput full-length 16S rRNA gene sequencing

## Abstract

Metagenomic studies of the Gouda cheese microbiota and starter cultures are scarce. During the present study, short-read metagenomic sequencing (Illumina) was applied on 89 Gouda cheese and processed milk samples, which have been investigated before concerning their metabolite and taxonomic composition, the latter applying amplicon-based, high-throughput sequencing (HTS) of the full-length 16S rRNA gene. Selected samples were additionally investigated using long-read metagenomic sequencing (Oxford Nanopore Technologies, ONT). Whereas the species identified by amplicon-based HTS and metagenomic sequencing were identical, the relative abundances of the major species differed significantly. *Lactococcus cremoris* was more abundant in the metagenomics-based taxonomic analysis compared to the amplicon-based one, whereas the opposite was true for the non-starter lactic acid bacteria (NSLAB). This discrepancy was related to a higher fragmentation of the lactococcal DNA compared with the DNA of other species when applying ONT. Possibly, a higher fragmentation was linked with a higher percentage of dead or metabolically inactive cells, suggesting that full-length 16S rRNA gene amplicon-based HTS might give a more accurate view on active cells. Further, fungi were not abundantly present in the Gouda cheeses examined, whereas about 2% of the metagenomic sequence reads was related to phages, with higher relative abundances in the cheese rinds and long-ripened cheeses. Intraspecies differences found by short-read metagenomic sequencing were in agreement with the amplicon sequence variants obtained previously, confirming the ability of full-length 16S rRNA gene amplicon-based HTS to reach a taxonomic assignment below species level. Metagenome-assembled genomes (MAGs) were retrieved for 15 species, among which the starter cultures *Lc. cremoris* and *Lactococcus lactis* and the NSLAB *Lacticaseibacillus paracasei*, *Loigolactobacillus rennini,* and *Tetragenococcus halophilus*, although obtaining MAGs from *Lc. cremoris* and *Lc. lactis* was more challenging because of a high intraspecies diversity and high similarity between these species. Long-read metagenomic sequencing could not improve the retrieval of lactococcal MAGs, but, overall, MAGs obtained by long-read metagenomic sequencing solely were superior compared with those obtained by short-read metagenomic sequencing solely, reaching a high-quality draft status of the genomes.

## 1 Introduction

The application of second-generation, high-throughput sequencing (HTS) has been a major breakthrough in cheese microbiology (Afshari et al., 2020; Parente et al., 2020; Zotta et al., 2022). It has considerably increased the research on cheeses (Quigley et al., 2012), and it has allowed the detection and quantification of sub-dominant and difficult-to-cultivate microorganisms, compared with culture-dependent approaches (Yeluri Jonnala et al., 2018). The importance of sub-dominant bacteria that do not grow on commonly used agar media in cheese studies has been demonstrated by tackling cheese spoilage problems, such as pink discoloration (Quigley et al., 2016) and crack formation (Decadt et al., 2024a). A widely used HTS approach during the past decade is amplicon-based sequencing of a part of the 16S rRNA gene, an approach that usually limits the taxonomic resolution to genus level (Parente et al., 2020). In contrast, shotgun metagenomics has a higher taxonomic resolution and accuracy compared with amplicon-based partial 16S rRNA gene HTS, and, in addition, it allows the analysis of the functional potential of the microbiota (Brumfield et al., 2020; Durazzi et al., 2021; Stothart et al., 2023). It is, however, more expensive and requires more sophisticated data processing approaches (Sedlar et al., 2017; Hillmann et al., 2018).

Recently, third-generation sequencing (TGS) has become mainstream. The TGS platforms of PacBio and Oxford Nanopore Technologies (ONT) allow to obtain read lengths up to 25 kbp and higher, as the read length of ONT is theoretically unlimited (van Dijk et al., 2023). Their main drawback is the higher error rate compared with Illumina short-read sequencing. TGS makes amplicon-based sequencing of the full-length 16S rRNA gene possible, increasing the taxonomic resolution to species level or lower, which is enhanced by the use of algorithms that result in amplicon sequence variants (ASVs) (Callahan et al., 2019; Decadt et al., 2023). For shotgun metagenomics, the long reads obtained by TGS allow more complete genome assemblies, higher numbers of retrieved metagenome-assembled genomes (MAGs), and increased taxonomic classification accuracy, compared with short-read sequencing (van Dijk et al., 2018; Pearman et al., 2020; Sereika et al., 2022).

The Gouda cheese microbiota can be divided into starter lactic acid bacteria (SLAB) and non-starter lactic bacteria (NSLAB). The SLAB consist of strains of *Lactococcus cremoris*, *Lactococcus lactis*, and one or more *Leuconostoc* species (e.g., *Leuconostoc mesenteroides* and *Leuconostoc pseudomesenteroides*), whereas the NSLAB consist of strains of *Lacticaseibacillus paracasei* and *Lactiplantibacillus plantarum* (Decadt and De Vuyst, 2023) but also *Loigolactobacillus rennini* and *Tetragenococcus halophilus* (Decadt et al., 2023, 2024a, 2024b). Hence, the species diversity of Gouda cheese is rather low, but the starter cultures applied are undefined and harbor a considerable intraspecies diversity (Smid et al., 2014). They contain different lineages of *Lc. cremoris* and *Lc. lactis*, which differ in phage and plasmid profiles (Erkus et al., 2013). It is, however, unclear how these *Lactococcus* lineages present in a Gouda cheese starter culture differ in flavor contribution. Among various Irish artisan cheeses, significant intraspecies differences of the SLAB *Lc. lactis* and *Streptococcus thermophilus* have been described, affecting the cheese volatilome (Walsh et al., 2020). However, the opposite has also been found. For instance, the four genetic lineages of *S. thermophilus* in an undefined thermophilic Swiss hard cheese starter culture are functionally redundant regarding their volatilome, and strains mainly differ in phage resistance potential (Somerville et al., 2022). To the authors’ knowledge, intraspecies diversity of the Gouda cheese microbiota and starter cultures has not yet been investigated using shotgun metagenomic sequencing (Decadt and De Vuyst, 2023).

The aim of the current study was to set up a methodology to obtain a more detailed insight into the Gouda cheese production chain and its microbiota, containing SLAB and NSLAB, by investigating 89 Gouda cheese and processed milk samples using a short-read, shotgun metagenomic sequencing approach, and to compare these results with taxonomic information previously obtained by amplicon-based full-length 16S rRNA gene HTS. Also, it aimed to retrieve MAGs using the short-read metagenomic sequencing datasets, MAGs using long-read metagenomic sequencing datasets for a selected number of samples, and MAGs using both types of sequencing dataset. Further, a methodology was set up to compare the MAGs obtained by both sequencing strategies as to assess which strategy allowed to obtain multiple MAGs for one species, given the typical starter culture composition, enhancing an intraspecies metagenomic approach.

## 2 Materials

### 2.1 Samples sequenced and analyzed

In the current study, 89 whole-community DNA samples, consisting of three groups of samples discussed in three previous studies on Gouda cheeses, were subjected to shotgun metagenomic sequencing (Table 1). Each sample was sequenced once. In those previous studies, the samples were analyzed for their metabolite and taxonomic composition, the latter by applying full-length 16S rRNA gene amplicon-based HTS (BioProject accession numbers PRJEB58546, PRJEB64168, and PRJEB64331). All samples were obtained from the same European Gouda cheese company, which uses three commercial starter cultures in rotation.

**Table 1:** Overview of the samples codes of the 89 Gouda cheese and processed milk samples sequenced.

Samples (34) from Gouda cheeses with batch-to-batch variability	
36 weeks of ripening, starter culture mixture A	A1_36 – A8_36
36 weeks of ripening, starter culture mixture B	B1_36 – B7_36
36 weeks of ripening, starter culture mixture C	C1_36 – C8_36
75 weeks of ripening, starter culture mixture A	A2_75, A7_75, A8_75
75 weeks of ripening, starter culture mixture B	B1_75, B5_75, B6_75, B7_75
75 weeks of ripening, starter culture mixture C	C3_75, C4_75, C7_75, C8_75

Samples (19) from Gouda cheeses with crack defects	
Zones with cracks	M1a_Cr, M1b_Cr, M1c_Cr, M2_Cr, L1a_Cr, L1b_Cr, L1c_Cr, L2_Cr, L3_Cr, XL_Cr
Zones without cracks	M1a_Z, M1b_Z, M1c_Z, M2_Z, L1a_Z, L1b_Z, L1c_Z, L2_Z, L3_Z

Samples (36) from a longitudinal study of a Gouda cheese production batch	
Thermized milk	0.0 h
Pasteurized milk after starter addition	0.9 h
Curd after cutting	1.8 h C
Whey after cutting	1.8 h W
Whey after washing	3.0 h W*
Curd after draining	3.7 h
Curd before brining	7.5 h
Cheese after brining	3 d
Cheese core (C) and rind (R) at 2, 4, 8, 12, 18, 25, 31, 36, 40, 45, 55, 65, 75, 85, and 100 weeks of ripening	2 w C, 2 w R – 100 w C, 100 w R

For the cheese samples with batch-to-batch variability, the letters A, B, and C refer to the starter culture mixture used, followed by a number referring to the different cheese production batches per starter culture, followed by a number referring to the duration of the ripening (in weeks). For the cheeses with crack defects, M, L, and XL refer to the severity of a crack defect (medium, large, and extra large, respectively). The numbers indicate different cheese production batches, whereas small letters (a, b, and c) indicate different cheese wheels that were investigated of the same Gouda cheese production batch. The samples from the longitudinal study of a Gouda cheese production batch, from thermized milk to long-ripened cheeses, are indicated by their time point during the production process. *, only sequenced using long-read metagenomic sequencing.

The first group of samples (34 in total) consisted of the cores of 23 Gouda cheeses obtained from different production batches that were made with three different starter culture mixtures and that showed batch-to-batch variability in organoleptic quality, even when the same starter culture was used (Table 1; Decadt et al., 2023). For all 23 production batches, cheese cores were collected after 36 weeks of ripening (ready for sale), and for 11 of these cheese production batches, cheese cores were also collected after 75 weeks of ripening (long-ripened cheese). The letters A, B, and C in the sample codes indicate the starter culture mixture used, whereas the numbers refer to the different cheese production batches per starter culture.

The second group of samples (19 in total) were obtained from six Gouda cheese production batches, for which the cheeses showed a crack defect after 31 weeks of ripening (Table 1; Decadt et al., 2024a). The letters in the sample codes refer to the severity of the crack defect, namely M for medium (two batches), L for large (three batches), and XL for extra large (one batch), whereas the numbers indicate different cheese production batches. For production batches M1 and L1, three different cheese wheels were investigated, indicated with the letters a, b, and c. From each cheese wheel, two samples of the core were taken, namely, one sample near the crack zones, and one from zones away from the cracks. However, cheese wheel XL had no zones without cracks, resulting in a total of 10 samples obtained from crack zones, and nine samples obtained from zones without cracks.

The third group of samples (36 in total) was collected during a longitudinal study of one Gouda cheese production batch, made with starter culture mixture A. Samples were collected at various key moments during the production process, from the thermized milk at the start of the production up to 100 weeks of cheese ripening (Table 1; Decadt et al., 2024b). During ripening, which started at week 2 and lasted until week 100, separate samples were taken from the cheese core (indicated by the letter C) and the cheese rind (indicated by the letter R), except for the time point after 2 weeks of ripening, at which only the core was sampled. As such, 15 core samples and 14 rind samples were obtained during the ripening period, in addition to eight samples from the production process itself (Table 1).

## 3 Methods

### 3.1 Short-read metagenomic sequencing

Whole-community DNA of the 89 samples targeted in this study was already obtained in the three previous studies, where it was used for full-length 16S rRNA gene amplicon-based HTS (Decadt et al., 2023, 2024a, 2024b). In the current study, the same samples of community DNA were used for short-read and long-read metagenomic sequencing. The DNA extraction method used was based on enzymatic digestion (using lyticase, Zymolyase, lysozyme, and mutanolysin), a chemical/enzymatic treatment (with sodium dodecyl sulfate and proteinase K), and a mechanical disruption (with acid-washed glass beads), followed by protein removal using a mixture of chloroform, phenol and isoamyl alcohol, an RNase treatment, and DNA purification with a DNeasy Blood & Tissue Kit (Qiagen, Venlo, The Netherlands).

The whole-community DNA samples were sequenced using an Illumina NovaSeq platform, applying a 2 × 250 bp paired-end sequencing approach and using a 500–1,500 bp fragment library (VUB-ULB BRIGHTcore sequencing facility, Jette, Belgium). Each sample was sequenced once. The quality of the metagenomic sequence reads (MSRs) obtained was visually assessed with FastQC (version 0.11.9; Andrews, 2018), followed by quality filtering and trimming using Trimmomatic (version 0.39; Bolger et al., 2014). For adapter trimming, the minimum adapter length was set to 2, the required match accuracy between two ligated adapters or between any adapter sequence and a read was set to 30 and 10, respectively, and a maximum mismatch count of 2 was used. The first 12 bases of the reads were removed, the minimal mean quality score was set to 20 over a sliding window of 4 bases, and the minimum quality score at both ends of the MSRs was set to 20. The minimum MSR length was set to 50 bases.

The forward and reverse high-quality paired-end reads obtained with Trimmomatic were merged using PANDAseq (version 2.11; Masella et al., 2012), with the minimum overlap set to 10 bases. The merged sequences obtained were subsequently pooled with the forward unpaired sequences from both Trimmomatic and PANDAseq, and with the reverse unpaired sequences from Trimmomatic, yielding 89 high-quality MSR (HQ-MSR) datasets.

### 3.2 Taxonomic identification based on short-read metagenomic sequence reads

Taxonomic identification of all HQ-MSRs was determined at three taxonomic levels, namely, genus, species, and intraspecies.

#### 3.2.1 Genus level

To perform taxonomic identification at genus level, three alignment-based approaches were used. The HQ-MSRs were aligned to the non-redundant protein database [nr; National Center for Biotechnology Information (NCBI), Bethesda, Maryland, United States] using DIAMOND (Buchfink et al., 2015), after which the output was further analyzed using MEGAN (Huson et al., 2016) with the lowest common ancestor (LCA) minimum score set to 100. In addition, the HQ-MSRs were analyzed using Kaiju, relying on a dedicated database that was developed using the nr protein database (Menzel et al., 2016). Furthermore, the HQ-MSRs were aligned to the non-redundant nucleotide database (nt; NCBI), to a subset of the RefSeq nucleotide database (NCBI), containing viral, bacterial, archaeal, and lower eukaryotic sequences, and to a similar subset of the RefSeq protein database (NCBI) using Kraken2 (Wood et al., 2019). The combination of different tools and databases allowed a software- and database-independent identification of the HQ-MSRs (Vermote et al., 2022). To verify if the combination of Kraken2 with the RefSeq nucleotide and the nt databases could identify viruses correctly, 308 *Skunavirus* sequences were downloaded from the NCBI Virus database and aligned to a subset of the RefSeq nucleotide database, as well as to a subset of the RefSeq protein database, using Kraken2.

#### 3.2.2 Species level

To perform taxonomic identification at species level, metagenomic recruitment plotting was applied. This method is based on the alignment of the HQ-MSRs to reference genomes to evaluate whether the corresponding species is present in the sample sequenced (Vermote et al., 2018). The evaluation is based on the application of minimum values for sequence identity and sequence coverage (see below), and the resulting plots are visually inspected. Before doing so, the 36 HQ-MSR datasets of the samples from the longitudinal study of a Gouda cheese production batch were aligned to the *Bos taurus* (Hereford) genome sequence (GCA_002263795.2) using blastn (Altschul et al., 1990). Sequence reads with a sequence identity and query coverage higher than 60% were deleted from the HQ-MSR datasets, as they were assumed to belong to *Bos taurus* and thus not from a microbial origin.

Next, the actual metagenomic recruitment plotting was performed for all 89 HQ-MSR datasets. As a first and preparatory step, a custom BLAST database was constructed, based on the output of the tools used for the taxonomic analysis at genus level. This database contained genome sequences of the type strains of all species belonging to those genera that had more than 0.1% of all sequences assigned to them. In the case no genome sequence was available for the type strain or the genome sequence was unreliable due to contamination, a genome sequence of another strain of the same species with the highest assembly level was used (Vermote et al., 2018). Next, the 89 HQ-MSR datasets were aligned to this custom BLAST database using blastn. The output was filtered using an in-house R script, which omitted all reads having a sequence identity lower than 60% and a sequence overlap shorter than 50 bases. The script also considered only the best hit, or randomly selected between the best hits in the case that several hits had the same highest bit score. The sequence alignment results obtained were subsequently plotted for all species that had more than 300 HQ-MSRs assigned to it using an in-house R script. All plots were manually checked and only the species with HQ-MSRs spread over the whole genome length were retained, as illustrated in Supplementary Figure S1, as this is an indication that the related species was present in the sample sequenced. Finally, for each species retained, the aligned HQ-MSRs were counted and expressed as relative abundances of all HQ-MSRs for all species retained.

#### 3.2.3 Intraspecies level

Whereas metagenomic recruitment plotting was used for taxonomic identification at the species level, this approach allowed in some cases to obtain insights into the intraspecies level too. Hereto, genomes of different subspecies were included in the custom BLAST database, namely, *Lc. cremoris* subsp. *cremoris*, *Lc. cremoris* subsp. *tructae*, *Lc. lactis* subsp. *hordniae*, *Lc. lactis* subsp. *lactis*, *Leuc. mesenteroides* subsp. *cremoris*, *Leuc. mesenteroides* subsp. *dextranicum*, *Leuc. mesenteroides* subsp. *jonggajibkimchii*, *Leuc. mesenteroides* subsp. *mesenteroides*, *Leuc. mesenteroides* subsp. *sake*, *Lacc. paracasei* subsp. *paracasei*, *Lacc. paracasei* subsp. *tolerans*, *T. halophilus* subsp. *halophilus*, and *T. halophilus* subsp. *flandriensis*. Additionally, the type strain of *Weissella jogaejeotgali* was included as well, although this species is currently considered as a synonym of *Weissella thailandensis* (Kwak et al., 2019). The ratio of the total number of reads recruited by multiple genomes within the same species was calculated for all 89 HQ-MSR datasets examined, and the average and standard deviations of all ratios were reported. Another way of identification below species level was done by expert inspection of the metagenomic recruitment plots.

In addition, StrainPhlAn3 (Truong et al., 2017) was used to assess strain-level differences among the 34 samples from the cheeses with batch-to-batch variability and the 19 samples from the cheeses with crack defects. Hereto, the HQ-MSRs were mapped to a database of species-specific marker genes (mpa_v30, accessed January 2021) using the companion tool MetaPhlAn3 (v3.0.13, Beghini et al., 2021). Then, all marker gene sequences were used to construct consensus marker gene sequences for each sample. The marker genes for *Lacp. plantarum*, *Leuc. pseudomesenteroides*, *Loil. rennini*, and *T. halophilus* were extracted and used to construct the phylogeny in the phylophlan accurate mode. The database still considered *Lc. cremoris* and *Lc. lactis* as the same species and, hence, only a joint phylogeny for both species could be obtained. The database did not contain marker genes for *Lacc. paracasei* and, hence, those were added manually. Hereto, a pangenome of *Lacc. paracasei* was constructed in anvi’o (Eren et al., 2015), using 53 genomes. To identify all single-copy core genes only found in this species, 25 genomes of lacticaseibacilli other than *Lacc. paracasei* were added, as well as 17 genomes of different lactiplantibacilli. Additionally, one genome of *Lactococcus laudensis, Lc. cremoris, Lc. lactis, Lentilactobacillus buchneri, Leuc. pseudomesenteroides, Limosilactobacillus fermentum, Loil. rennini,* and *T. halophilus* were added, representing more distinctly related LAB species, of which most were found in the cheese samples. A total of 478 single-copy core genes was found, of which 110 were randomly taken from *Lacc. paracasei* Zhang and added to the mpa_v30 database. The final phylogenetic trees were visualized using the R package ggtree (Yu et al., 2017).

### 3.3 Long-read metagenomic sequencing

Long-read metagenomic sequencing was performed using whole-community DNA of four samples from the longitudinal study of a production batch, namely 3.0 h W, 18 w C, 45 w C, and 45 w R (Table 1), using the ONT’s MinION sequencer (Oxford, United Kingdom). Sample 3.0 h W was only sequenced using long-read metagenomic sequencing because there was too little DNA available to also sequence it using short-read metagenomic sequencing. For each sample, 1 μg of whole-community DNA samples (same aliquots as were used for full-length 16S rRNA gene amplicon-based HTS in previous studies and short-read metagenomic sequencing in the current study) was used to prepare the DNA library using the SQK-LSK110 Ligation Sequencing kit (ONT), following the manufacturer’s instructions. The final DNA library was loaded on an R9.4.1 FLO-MIN106 flow cell. For each sample, one flow cell was used. Basecalling of the raw sequencing signal was performed using Guppy (v6.0.6; ONT). Only the reads classified as “pass” by Guppy (Qscore >9) were considered for any downstream analysis. Trimming was performed using NanoFilt (v2.8.0; De Coster et al., 2018) to remove the adapters and low-quality bases at both extremities of the reads (headcrop, 40; tailcrop, 20) and to discard the reads shorter than 500 bp.

### 3.4 Taxonomic identification based on long-read metagenomic sequence reads

Metagenomic recruitment plotting was performed to taxonomically identify the HQ-MSRs obtained at species level, similarly as described above. Briefly, all long-read HQ-MSRs were aligned to the same custom BLAST database as used for the short-read HQ-MSRs using blastn. The output was filtered using the same in-house filtering R script as mentioned above, and omitting all reads having a sequence identity lower than 70%. The sequence alignment results obtained were subsequently plotted using an in-house R script, optimized for long reads. Plots of all species were manually checked and species without well-spread reads were rejected as illustrated in Supplementary Figure S2, using a similar strategy as for the short-read metagenomic recruitment plotting.

Finally, for each species that was retained, the sum of all bases of all reads recruited was considered and expressed as relative abundances compared to the sum of all bases of all reads of all species retained. In this way, the variable length of the reads was also taken into account, in contrast to the short-read sequencing approach, for which all reads had a comparable short length and the count of the reads was as such reliable. A second analysis was performed by omitting all reads shorter than a certain threshold read length. The threshold was set to values between 1 and 10 kbp. Distributions of the read lengths per species were plotted with the geom_density function in ggplot2 (version 3.3.5; Wickham, 2016).

Based on the results of the long-read metagenomic sequencing, so far unpublished sequence data of high-throughput amplicon-based HTS of the V4 region of the 16S rRNA gene for two Gouda cheese samples (the cores of the batch A1 cheeses taken after 26 and 31 weeks of ripening) were considered in the current study as well. The amplification and sequencing were carried out as described previously, using the primer set F515/R806 (De Bruyn et al., 2017).

### 3.5 Metagenome-assembled genome reconstruction

#### 3.5.1 Assembly, binning, and functional analysis of short-read metagenomic sequences

For the assembly and binning, the 89 HQ-MSR datasets were divided over five subsets, namely, one subset containing all datasets of samples from the longitudinal study of a Gouda cheese production batch, one subset containing all datasets of the samples from Gouda cheeses with crack defects, and three subsets containing the datasets of the samples from Gouda cheeses with batch-to-batch variability, divided per starter culture mixture used. For all five subsets, all HQ-MSRs were co-assembled using MEGAHIT (version 1.2.9; Li et al., 2015) with a minimum contig length of 1,000 bases. The resulting contigs were further filtered to a minimum length of 2,500 bp using anvi’o, a comprehensive analysis platform combining cutting-edge computational strategies for data-enabled microbiology (Eren et al., 2015). Taxonomic profiling of the contigs was performed by classification of the anvi’o-generated predicted genes using Kaiju. Next, the HQ-MSRs of each dataset were mapped to the co-assembly obtained using Bowtie2 (Langmead and Salzberg, 2012), resulting in an anvi’o profile for each HQ-MSR dataset. These anvi’o profiles were merged into one anvi’o profile database, and the profiles obtained were subsequently binned using CONCOCT (Alneberg et al., 2014). Next, only bins with a completion of more than 75% were manually refined, based on a stable G + C content and consistent read coverage, to be able to obtain bins with a high completion (>75%) and low redundancy (<10%). The resulting bins are further referred to as MAGs.

In addition, and based on the results obtained, a selection of the 89 HQ-MSR datasets was divided into another series of five different subsets, and processed in the same way as described above. Three of these subsets contained only HQ-MSR datasets related to the cores of the Gouda cheese with batch-to-batch variability with a low relative abundance of *Lactococcus*, divided per starter culture mixture used. Two subsets contained HQ-MSR datasets of samples having one of the two main clusters of *T. halophilus* (Table 2).

**Table 2:** Overview of the five additional subsets, with the HQ-MSR datasets included in each subset for metagenome-assembled genome (MAG) retrieval.

Subset	Samples
Cheese cores with low relative abundance of *Lactococcus*	
Starter culture mixture A	A8_75, M2_Cr, L2_Cr, XL_Cr
Starter culture mixture B	B1_75, B7_75, L1a_Cr, L1b_Cr, L1c_Cr, L3_Cr
Starter culture mixture C	C3_75, C7_75, C8_75
*Tetragenococcus halophilus*	
Cluster 1	M1a_Cr, M1b_Cr, M1c_Cr, L3_Cr
Cluster 2	B1_75, B5_36, C4_75, C5_75, C7_75, C8_75

A, B, and C refer to the starter culture mixture used, whereas M, L, and XL refer to the severity of a crack defect (medium, large, and extra large, respectively). The numbers indicate different cheese production batches, whereas small letters (a, b, and c) indicate different cheese wheels of the same Gouda cheese production batch. 36w, ripened for 36 weeks; 75w, ripened for 75 weeks.

For each metagenomic bin, the species-level identification was determined using fastANI (v1.33; Jain et al., 2018) and a custom BLAST database made of whole-genome sequences of type strains for each species of the main genera found in all metagenomic samples, as described previously (Vermote et al., 2018). Functional annotation of the contigs of the MAGs was done using Prokka (version 1.14.6; Seemann, 2014), with the default settings, an e-value cut-off of 1 × 10^−20^, and addition of gene features for each coding DNA sequence (CDS). The output file was used to manually search for genes of interest, including 5S, 16S, and 23S rRNA genes and tRNA genes.

#### 3.5.2 Assembly, binning, and functional analysis of long-read metagenomic sequences

To assess the best methodology to reconstruct high-quality MAGs using short reads, long reads, or a combination of both, a comparison of different software tools was performed (Supplementary Figure S3). For a correct reconstruction, only the four short-read HQ-MSR datasets, corresponding with the four Gouda cheese samples of the long-read sequencing, were used. Sample 1.8 h W (sequenced with short-read sequencing solely) and sample 3.0 h W (sequenced with long-read sequencing solely) were considered as corresponding samples given the fact that these samples were very similar regarding microbial composition according to the results obtained previously (Decadt et al., 2024b). Unless stated otherwise, all tools were used with their default settings. The quality-filtered and trimmed short reads were used to perform a co-assembly using MEGAHIT (v1.2.9; Li et al., 2015; assembly further referred to as COM) with the “bubble level” set to 1, and metaSPAdes (v3.14.0; Nurk et al., 2017; assembly further referred to as COS) with the k-mer size set to 77. The short-read co-assemblies were curated with metaMIC (Lai et al., 2022), and are further referred to as COM.MIC and COS.MIC, respectively.

Quality-filtered and trimmed long reads were co-assembled using metaFlye (v2.9.1; Kolmogorov et al., 2020). Two different co-assemblies were generated, namely, one with the option to keep haplotypes (COFH), and one without that option (COF). To determine whether the incorporation of short reads would allow to correct a metagenome assembled using long reads only, and hence would improve subsequent downstream analysis, the COFH assembly was subjected to a polishing strategy, as is typically performed for genomes (Díaz-Muñoz et al., 2022). Briefly, four iterations of Racon (v1.4.21; Vaser et al., 2017) and one iteration of medaka (v1.5.0; ONT) were used to obtain more accurate consensus sequences, using only long reads. Next, the Illumina short reads were used to correct the co-assembly by means of Pilon (v1.24; Walker et al., 2014; COFHP). This co-assembly was further processed using Strainberry (v1.1; Vicedomini et al., 2021; COFHPB), aiming at achieving a higher haplotype resolution of the MAGs.

The eight different co-assemblies generated were used as input to perform an automatic metagenomic binning using CONCOCT (v1.1.0; Alneberg et al., 2014). The quality of the metagenomic bins was evaluated using CheckM (v1.2.2; Parks et al., 2015). Only metagenomic bins with at least 10% completeness were considered. The species-level identification was performed using fastANI (v1.33; Jain et al., 2018), as described above. If a bin could not be identified at species level using this method, it was identified at genus level using Kraken2 (v2.0.8; Wood et al., 2019). The 16S rRNA copy number obtained was compared with the information from the rrnDB database (Stoddard et al., 2015). To compare all MAGs obtained with the MAGs obtained using short-read metagenomic sequence reads (Section 3.5.1), the average nucleotide identity (ANI) and the full ANI values were calculated between each pair of the same species using pyANI (Pritchard et al., 2016). Finally, functional annotation of the MAGs (> 75% completion and < 10% redundancy) obtained by COM.MIC (considered as the best short-read method; this study), COFH (considered as the best long-read method; this study), and COFHP (considered as the best method combining long and short reads; this study) was done using Prokka (version 1.14.6; Seemann, 2014), as described above.

### 3.6 Statistics

A Spearman correlation was calculated between Gouda cheese ripening time and the relative abundances of viral and fungal DNA for both cheese core and rind samples of the longitudinal study of a production batch. Additionally, a paired *t*-test was performed between relative abundances of viruses and fungi in the cheese cores and rinds, and between the relative abundances of bacterial species found by short-read shotgun metagenomics compared with high-throughput, full-length 16S rRNA amplicon-based HTS obtained in previous studies. The results were considered significant when the *p*-value <0.05. All statistical analyses were performed in R (version 4.1.0; R Core Team, 2021).

## 4 Results

### 4.1 Taxonomic identification based on short-read metagenomic sequence reads

#### 4.1.1 Genus level

For the 89 HQ-MSR datasets obtained, five alignment-based approaches were applied for taxonomical identification at genus level. The two approaches using nucleotide-based identification (Kraken2 NCBI nt and Kraken2 RefSeq nucleotide) could identify more reads at genus level compared to the three approaches using protein-based identification (DIAMOND, Kaiju, and Kraken2 RefSeq protein) (Supplementary Figure S4). All five approaches identified *Lactococcus* as the most abundant genus in almost all samples examined. Exceptions were sample A8 (cheeses with batch-to-batch variability), and M2, L1b, and L2 (cheeses with crack defects), which had *Loigolactobacillus* as the most abundant genus in the case of DIAMOND (and Kaiju for L1b). Additionally, the rinds of the cheeses obtained after 65 and 100 weeks of ripening from the longitudinal study had *Tetragenococcus* as most abundant genus according to all approaches. The thermized milk (0.0 h) of the longitudinal study had *Mammalia* as most abundant taxonomic group according to Kraken2 NCBI nt, Kraken2 RefSeq nucleotide, and DIAMOND, whereas Kraken2 RefSeq protein and Kaiju resulted in *Babesia* and *Staphylococcus*, respectively, as the most abundant genera.

Besides *Lactococcus*, other abundant genera present in the Gouda cheese samples were *Tetragenococcus*, *Loigolactobacillus*, and *Leuconostoc*, except for the Kraken2 Refseq nucleotide approach that showed almost no *Loigolactobacillus*. Additionally, this approach did not detect viruses, and showed a higher relative abundance of *Bacillus* compared with the other approaches. This could be related to the database used, as all 308 *Skunavirus* sequences downloaded from the NCBI Virus database were identified as *Bacillus thuringiensis* by Kraken2 Refseq nucleotide, whereas all these sequences were correctly assigned to *Skunavirus* using Kraken2 Refseq protein.

According to Kraken2 NCBI nt, on average 1.9% of the reads were viral (median of 0.8%). More precisely, 50 samples had less than 1% viral reads, 21 samples had between 1 and 3% viral reads, 17 samples had between 3 and 8% viral reads, and one sample had 14.8% viral reads (Supplementary Table S1). More than 95% of these viral reads were assigned to *Skunavirus*, a lactococcal phage. The second and third most abundant viral genera, *Vedamuthuvirus* and *Sandinevirus*, were also lactococcal phages. In the samples from the Gouda cheeses with batch-to-batch variability, a higher relative abundance of viruses was found in the longest ripened cheeses (75 weeks) in seven out of 11 cheeses examined. Similarly, in the Gouda cheese samples from the longitudinal study, there was a significant (*p* < 0.05) increase in relative abundance of viral reads as a function of the ripening time. Additionally, the relative abundance of the viral reads was significantly higher in the cheese rinds compared to the cheese cores (*p* < 0.05).

Fungal reads were a minority, with an average relative abundance of 0.064% and a maximum relative abundance of 0.200% among all samples examined. The relative abundance of fungi did not significantly increase with the ripening time, neither was it different in the cheese cores compared to the rinds. The most abundant genus for all samples was *Saccharomyces* (on average 41.8% of all fungal reads), followed by *Debaryomyces* (on average 5.2% of all fungal reads).

#### 4.1.2 Species level

**Figure 1:**
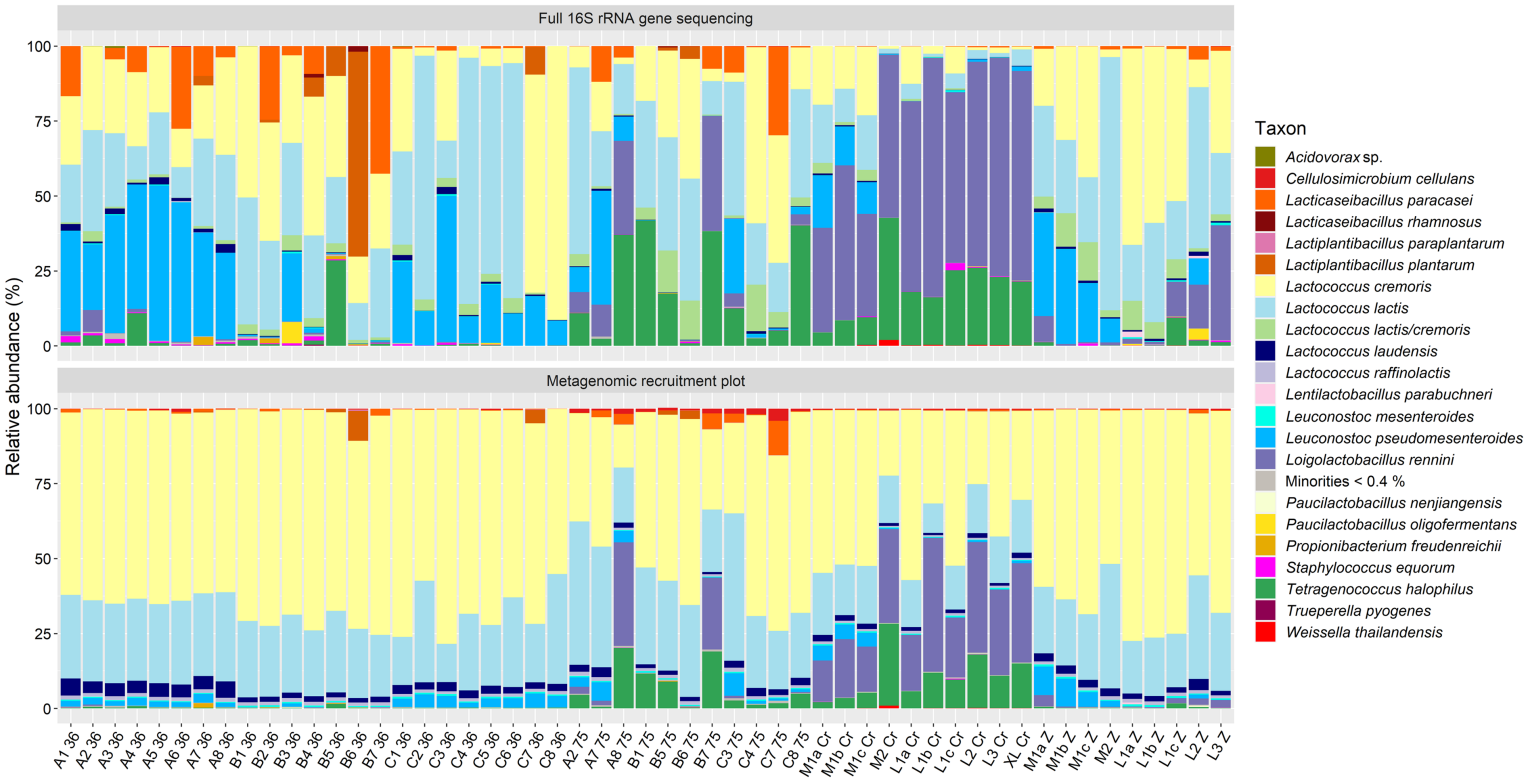
Taxonomic classification at the species level of the high-quality metagenomic sequence reads for the 23 Gouda cheeses with batch-to-batch variability and the cheeses with crack defects (names as explained in Table 1). Top, relative abundance based on high-throughput, full-length 16S rRNA amplicon-based sequencing, as reported previously (Decadt et al., 2023, 2024a). Bottom, relative abundance based on metagenomic recruitment plotting of high-quality metagenomic sequence reads; reads not assigned to any species are not shown. The category “Minorities” includes all species identified that were represented by less than 0.4% for both methods.

**Figure 2:**
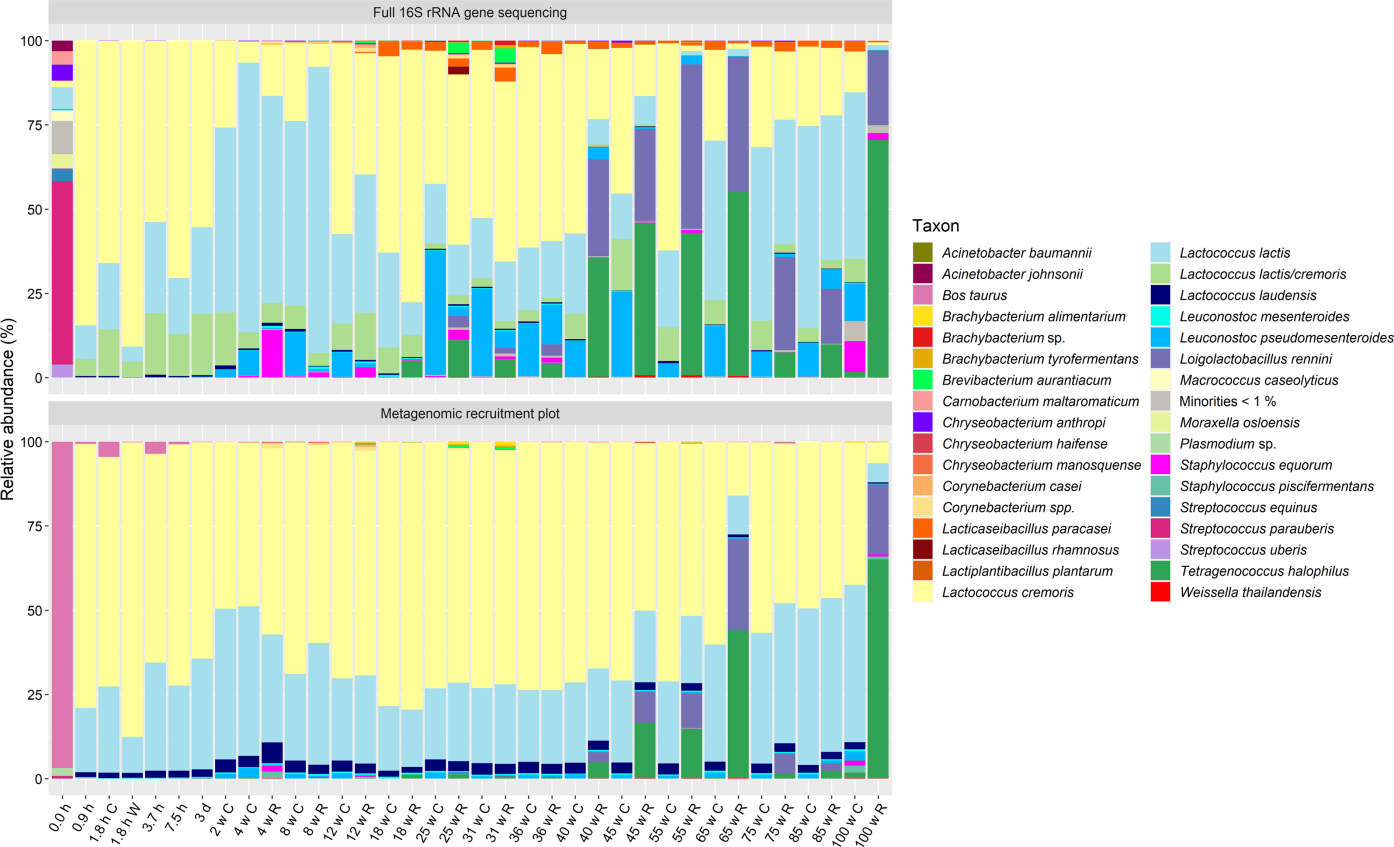
Taxonomic classification at the species level of the high-quality metagenomic sequence reads for the processed milk and cheese samples from the longitudinal study of a Gouda cheese production batch (C, curd/core; W, whey; R, rind). Top, relative abundance based on high-throughput, full-length 16S rRNA amplicon-based sequencing, as reported previously (Decadt et al., 2024b). Bottom, relative abundance based on metagenomic recruitment plotting of high-quality metagenomic sequence reads; reads not assigned to any species are not shown. The category “Minorities” includes all species identified that were represented by less than 1% for both methods.

The taxonomic identification at species level based on metagenomic recruitment plotting showed that *Lc. cremoris* was the most abundant species with an average relative abundance of 55.4% for all samples examined (standard deviation of 16.5%) (Figures 1, 2). *Lactococcus lactis* was the second most abundant species, with an average relative abundance of 24.0% for all samples (standard deviation of 8.8%). Other abundant species were *Loil. rennini* and *T. halophilus*, especially in the samples of cheeses with cracks and in the rind samples from the longitudinal study. *Lactococcus laudensis* and *Leuc. pseudomesenteroides* were present at lower relative abundances. The thermized milk sample (0.0 h) of the longitudinal study was odd compared to all other samples and contained mostly *B. taurus*.

Next, the species-level identification based on metagenomic recruitment plotting was compared with the data of species-level identification using high-throughput, full-length 16S rRNA amplicon-based HTS (Decadt et al., 2023, 2024a, 2024b) (Figures 1, 2). Although the species identified were the same in all samples (except for the thermized milk sample from the longitudinal study), the relative abundances differed between both methods. The relative abundances of *Lc. cremoris* and *Lc. laudensis* were significantly higher in the metagenomic recruitment plots compared with the amplicon-based HTS approach, whereas the opposite was true for most other species. Only the relative abundance of *Lc. lactis* was not significantly different between both methods.

#### 4.1.3 Intraspecies level

With respect to the intraspecies level, the number of reads obtained through metagenomic recruitment plotting and assigned to *Lc. cremoris* subsp. *cremoris* were approximately five times higher than those assigned to *Lc. cremoris* subsp. *tructae* in all samples examined (Supplementary Table S2). The same was the case for *Lc. lactis* subsp. *lactis* compared with the lesser abundant *Lc. lactis* subsp. *hordniae*. Additionally, *Leuc. mesenteroides* subsp. *cremoris* was also more abundant compared with all other *Leuc. mesenteroides* subspecies. Probably, *Lc. cremoris* subsp. *cremoris*, *Lc. lactis* subsp. *lactis*, and *Leuc. mesenteroides* subsp. *cremoris* were the subspecies present in the samples, and the other subspecies were not present but found by the metagenomic recruitment plotting because of high genetic similarities. *Lacticaseibacillus paracasei* subsp. *paracasei* was almost twice as abundant as *Lacc. paracasei* subsp. *tolerans*, suggesting it was foremost the former subspecies that was present. The number of reads recruited for both *T. halophilus* subspecies was very similar.

Additionally, the number of reads recruited for *Leuconostoc falkenbergense* and *Leuc. pseudomesenteroides* [reclassified as *Leuc. falkenbergense* by the genome taxonomy database (GTDB), but not yet by the NCBI Genome database at the moment of analysis] were very similar (ratio of 0.91). As a consequence of the undecided taxonomic status of the genomes used, and for comparison purposes with the 16S rRNA amplicon-based sequence data obtained, the reads of both species were summed and considered as *Leuc. pseudomesenteroides*. For some Gouda cheese samples, there was a gap consistently present at the same genomic location for *Leuc. pseudomesenteroides,* as depicted in the recruitment plot (Supplementary Figure S5). The same samples also had some gaps in the recruitment plot for *Leuc. falkenbergense.* All cheese samples with these characterizing gaps were from Gouda cheeses made with starter culture mixture C, whereas all cheese samples without these gaps were from Gouda cheeses made with starter culture mixtures A or B, although Gouda cheeses made with starter culture mixture B had almost no *Leuconostoc* reads (Decadt et al., 2023). There were two Gouda cheese batches that deviated from this trend, namely, sample C3 from the cheeses with batch-to-batch variability had no gaps, such as the Gouda cheeses made with starter culture mixture A, and sample M1 from the cheeses with cracks defects made with starter culture mixture A, had gaps compared to the Gouda cheeses made with starter culture mixture C. The recruitment plots for *Loil. rennini* also showed some gaps (Supplementary Figure S6), but the gaps were consistently at the same locations in the genomes for all samples, suggesting all *Loil. rennini* strains in the Gouda cheese ecosystem of the present study deviated in the same way from the reference strain. Additionally, five times more reads were recruited for the genome of *W. jogaejeotgali* compared with *W. thailandensis* (Supplementary Table S2), which suggested that both genomes differed significantly and that the strain(s) in the Gouda cheese ecosystem of the present study were more alike the *W. jogaejeotgali* genome (Supplementary Figure S7).

Next, the most prevalent bacterial species were investigated using StrainPhlAn. The analysis of *Leuc. mesenteroides*, *Staphylococcus equorum*, and *Lacticaseibacillus rhamnosus* failed because of a lack of reads for these species. Owing to the relatively recent elevation of *Lc. cremoris* to the species level, after being a *Lc. lactis* subspecies (Li et al., 2021), the available database of species-specific marker genes was not updated yet. The main difference between the Gouda cheese samples was, therefore, species-based, and related to the cheese age. Of the cheeses with the study on batch-to-batch variability, 20 of the 23 Gouda cheeses ripened for 36 weeks were part of the *Lc. cremoris* cluster, whereas eight of the 11 Gouda cheeses ripened for 75 weeks were part of the *Lc. lactis* cluster. Additionally, 14 of the 19 cheese samples of the study on crack defects clustered with *Lc. cremoris*, whereas the remaining five clustered with *Lc. lactis*. All Gouda cheese samples from the longitudinal study from 65 until 100 weeks of ripening were part of the *Lc. lactis* cluster, together with those after 0, 2, and 4 weeks of ripening, whereas the other samples clustered with *Lc. cremoris*.

**Figure 3:**
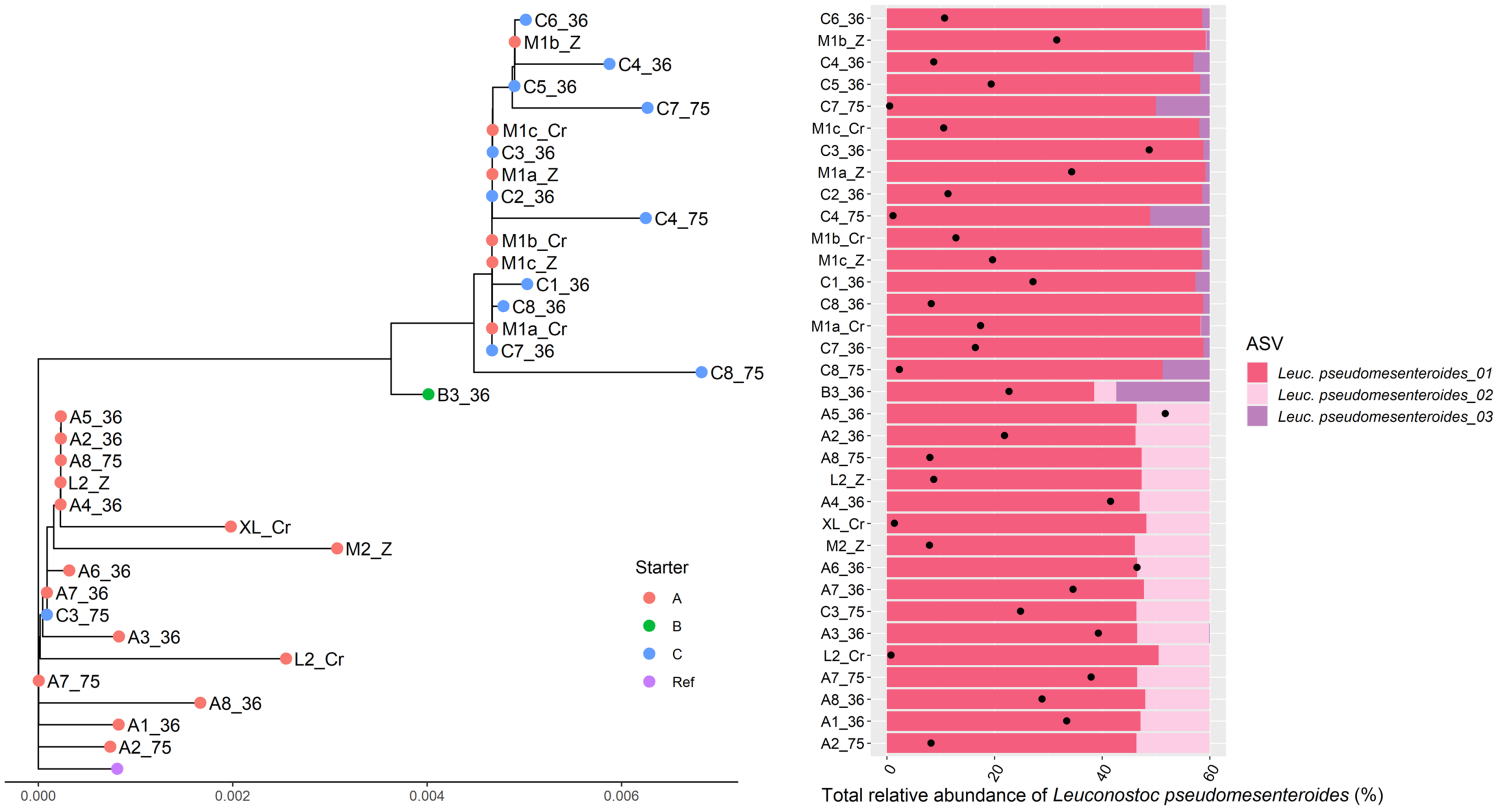
Intraspecies-level classification of the Gouda cheese starter culture species *Leuconostoc pseudomesenteroides* across the cheese samples examined. Left. Intraspecies-level phylogeny based on single nucleotide variants recovered from species-specific marker genes using StrainPhlAn3. Sample names are as explained in Table 1. Red dots, Gouda cheeses made with starter culture mixture A; green dots, Gouda cheeses made with starter culture mixture B; blue dots, Gouda cheeses made with starter culture mixture C; purple dot, reference genome of *Leuc. pseudomesenteroides* 4882. Right. Intraspecies-level diversity based on amplicon sequence variants (ASVs) obtained previously for the corresponding samples of the intraspecies-level phylogeny shown left (Decadt et al., 2023, 2024a). The colors represent the different ASVs found as well as their ratio. The black dots represent the total relative abundance of *Leuc. pseudomesenteroides* in the amplicon-based sequence data of each sample.

**Figure 4:**
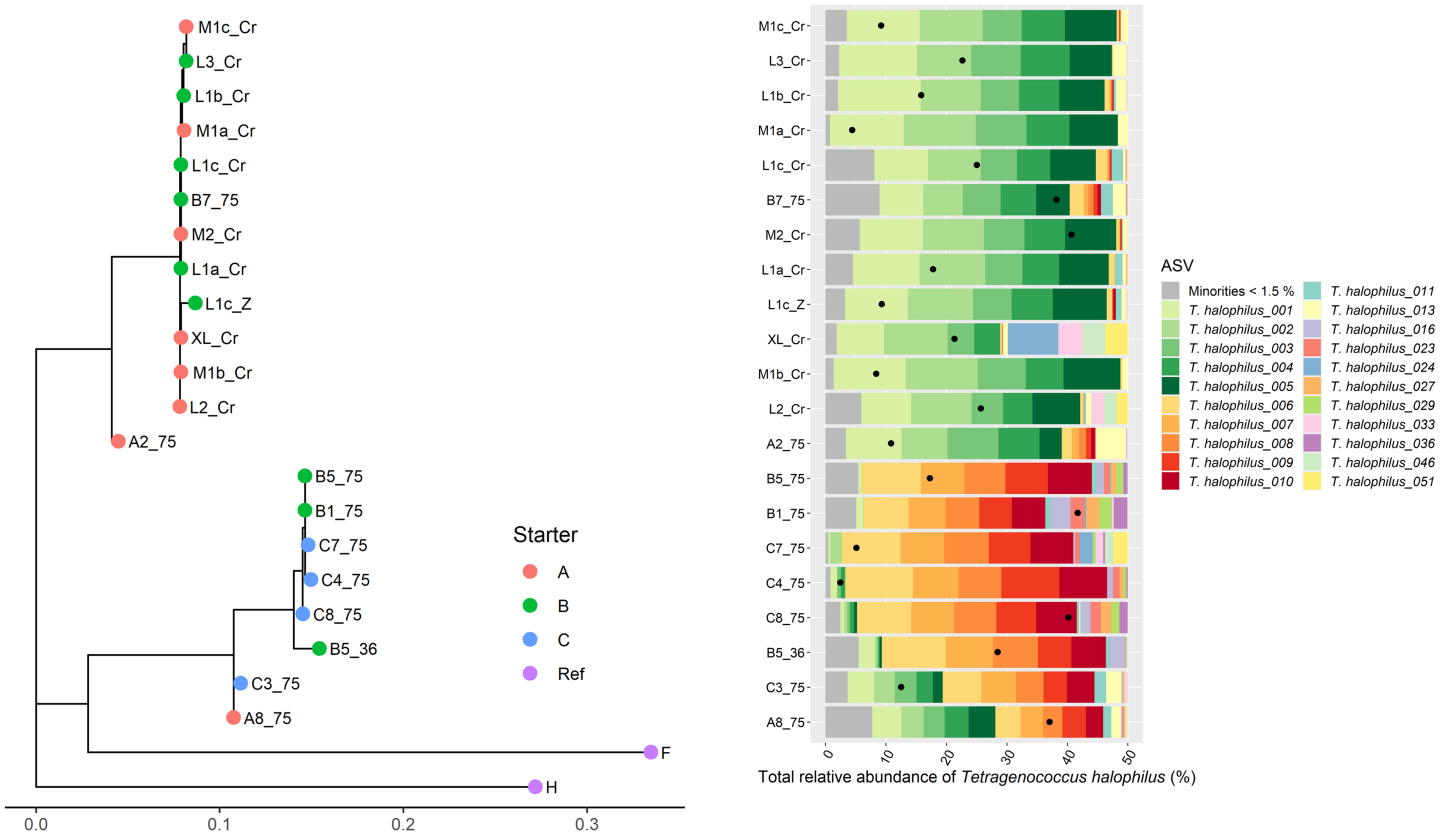
Intraspecies-level classification of *Tetragenococcus halophilus*. The description of the figure is analogous to Figure 3. Purple dots, reference genomes; F, *T. halophilus* subsp. *flandriensis* DSM 23766; H, *T. halophilus* subsp*. halophilus* DSM 20339. The category “Minorities” includes all ASVs that were represented by less than 1.5% of the total amplicon-based sequence reads in all samples.

For *Leuc. pseudomesenteroides*, two clusters could be distinguished across the Gouda cheese samples examined (Figure 3), which were in accordance with the starter culture mixtures used, with the same two exceptions as described above in the case of the metagenomic recruitment plots. The StrainPhlAn clustering was completely in line with the ASV profiles found previously (Decadt et al., 2023). Indeed, the *Leuc. pseudomesenteroides*_02 ASV was only found in one of the clusters, the *Leuc. pseudomesenteroides*_03 ASV only in the other cluster. One cheese sample (B3) was in between both clusters and had the ASVs of both clusters. Similarly, for *T. halophilus*, two main clusters coincided with two different ASV profiles as found previously (Decadt et al., 2023; Figure 4).

**Figure 5:**
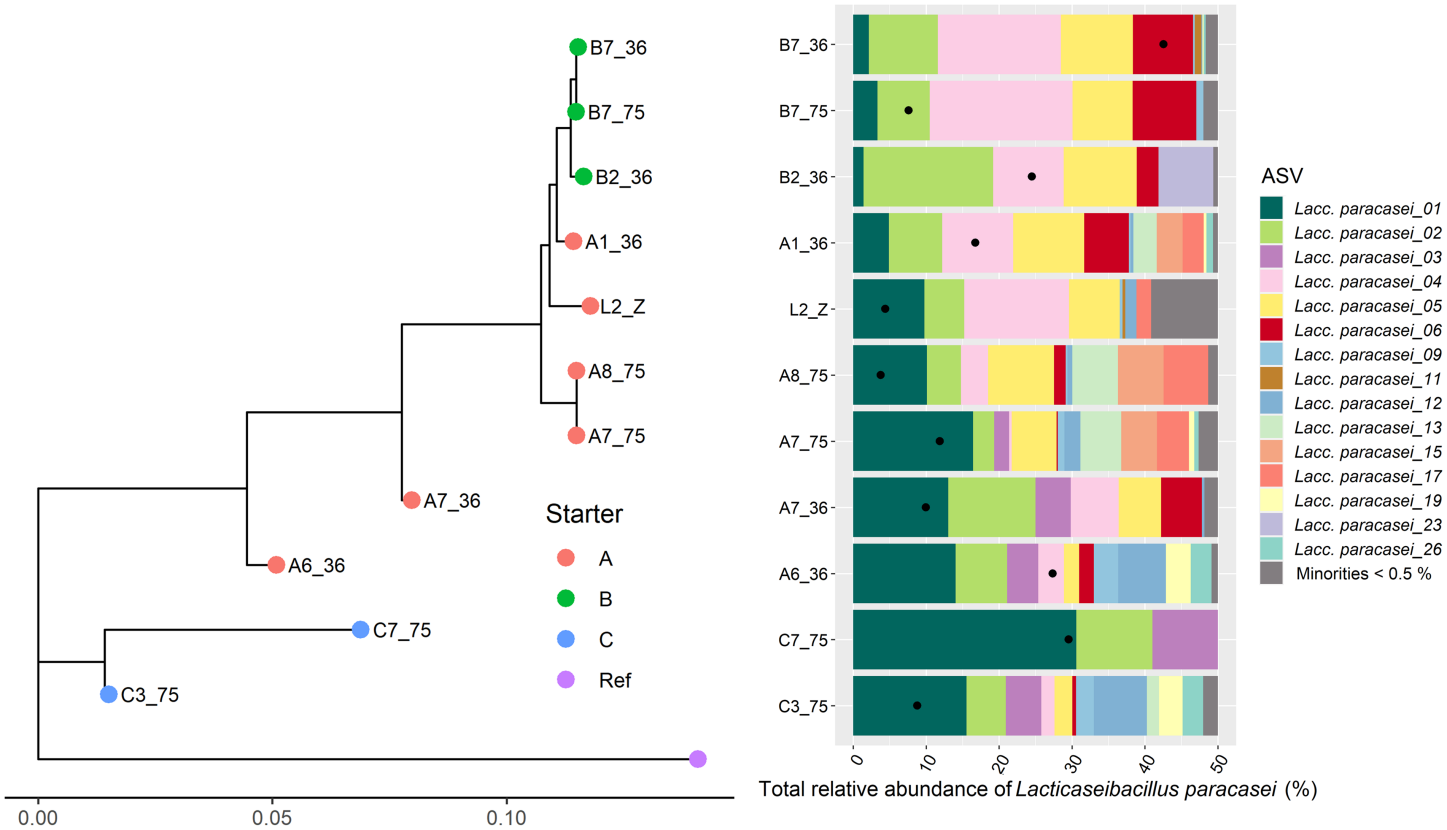
Intraspecies-level classification of *Lacticaseibacillus paracasei*. The description of the figure is analogous to Figure 3. Purple dot, reference genome of *Lacc. paracasei* Zhang ASM1924v3. The category “Minorities” includes all ASVs that were represented by less than 0.5% of the total amplicon-based sequence reads in all samples. The ASVs of samples without sufficient high-quality metagenomic sequence reads to obtain an intraspecies phylogeny using StrainPhlAn3 are not shown.

**Figure 6:**
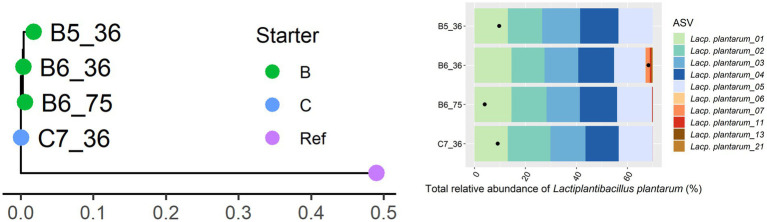
Intraspecies-level classification of *Lactiplantibacillus plantarum*. The description of the figure is analogous to Figure 3. Purple dot, reference genome of *Lacp. plantarum* subsp. *plantarum* ATCC 14917. The ASVs of samples without sufficient high-quality metagenomic sequence reads to obtain an intraspecies phylogeny using StrainPhlAn3 are not shown.

**Figure 7:**
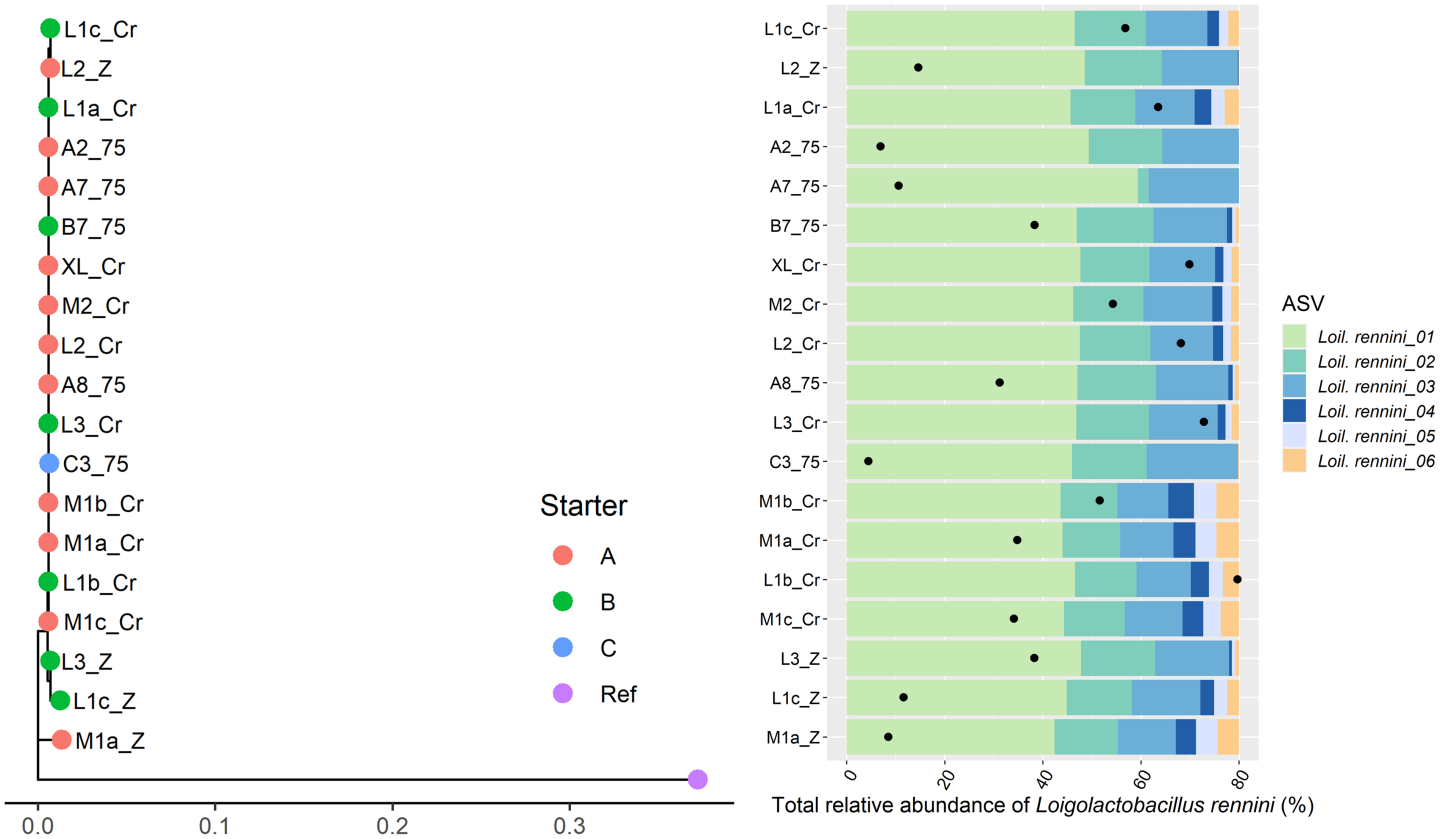
Intraspecies-level classification of *Loigolactobacillus rennini*. The description of the figure is analogous to Figure 3. Purple dot, reference genome of *Loil. rennini* DSM 20253. The ASVs of samples without sufficient high-quality metagenomic sequence reads to obtain an intraspecies phylogeny using StrainPhlAn3 are not shown.

Only 11 Gouda cheese samples had a sufficient number of sequence reads for *Lacc. paracasei* to be detected by StrainPhlAn (Figure 5). They were mainly clustered based on the starter culture mixtures used. In the case of *Lacp. plantarum* and *Loil. rennini*, all cheese samples were part of one cluster (Figures 6, 7).

### 4.2 Taxonomic identification based on long-read metagenomic sequence reads

**Figure 8:**
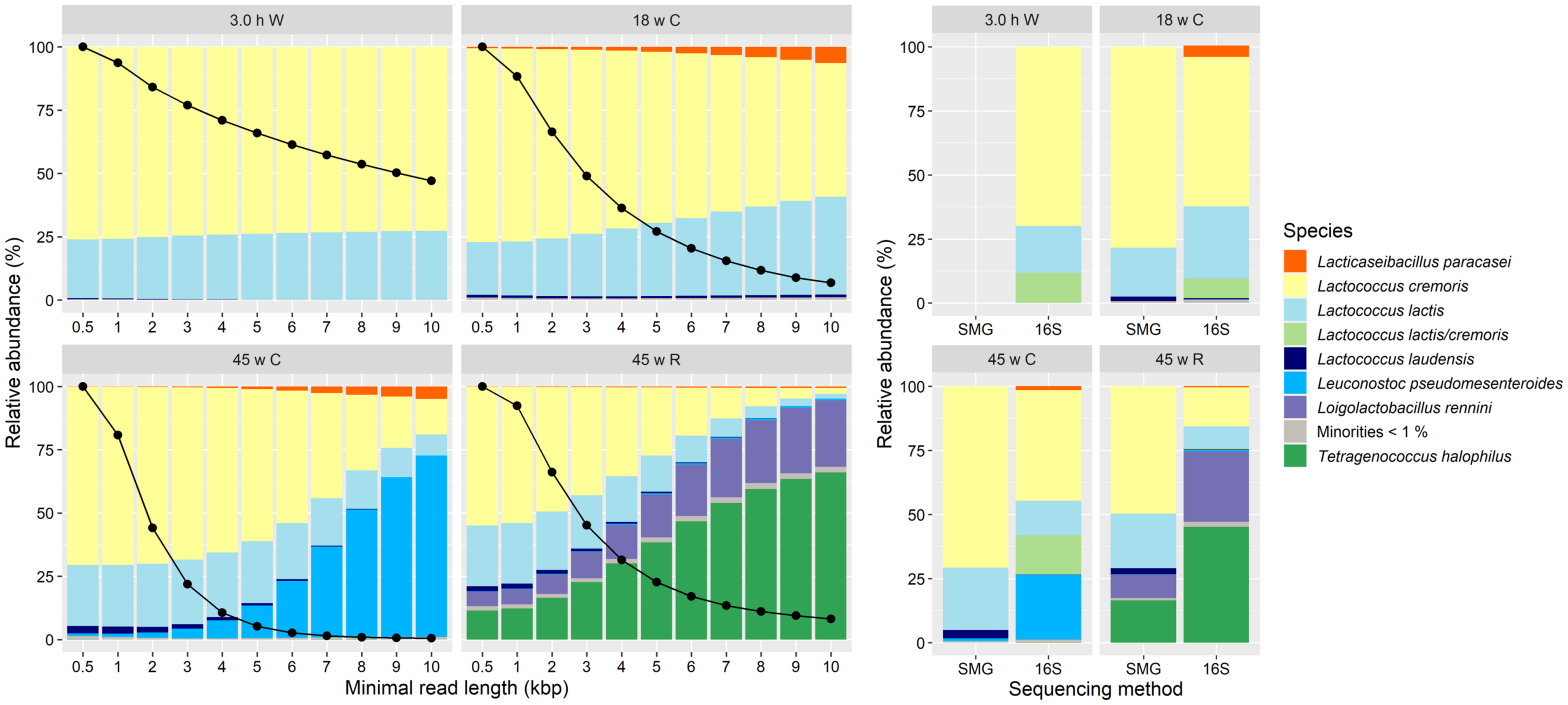
Taxonomic classification at the species level of all sequence reads for the cheeses from the longitudinal study of a Gouda cheese production batch. Left. Classification expressed as relative abundance of base pairs assigned to each species based on metagenomic recruitment plotting of long-read metagenomic sequencing. Reads not assigned to any species are not shown. By default, all reads shorter than 0.5 kbp were removed, but, additionally, higher minimal read lengths were used as a cut-off criterium, indicated on the *X*-axis. The black dots on the *Y*-axis represent the percentage of total base pairs retained by the increased minimal read length, compared to the default filtering. Right. Classification based on short-read metagenomic sequencing (SMG) and high-throughput, full-length 16S rRNA amplicon-based sequencing (16S). The category “Minorities” includes all species identified that were represented by less than 1%. W, whey; C, core; R, rind.

Taxonomic identification of the four Gouda cheese samples subjected to long-read metagenomic sequencing resulted in the same main species as those that were found with the short-read metagenomic sequencing and high-throughput, full-length 16S rRNA amplicon-based HTS approaches (Figure 8). Also, the relative abundances of these species were in agreement with those of the short-read sequencing data. However, when the minimal read length of the long-read sequences considered for taxonomic analysis was increased, these relative abundances were more in agreement with those obtained by high-throughput, full-length 16S rRNA amplicon-based HTS (Figure 8). The number of reads decreased with increasing minimal read length, but the number of reads assigned to *Lactococcus*, especially *Lc. cremoris*, decreased much faster compared with the number of reads assigned to other species. This was also illustrated by the density plots of the read length for each main species (Supplementary Figure S8). A minimal read length of 6 kbp resulted into the closest similarity of relative abundances compared to the amplicon-based sequencing data.

The species-dependent differences of the read length for long-read metagenomic sequencing raised the question if the relative abundances of taxa determined by amplicon-based HTS might also depend on the length of the amplicon sequence targeted. Indeed, to be amplified, the target sequence needs to be complete. Mathematically, the fraction *I* of intact target sequences with length *L* (300 bp for the V4 region and 1,500 bp for the full-length 16S rRNA gene) as a function of a hypothetically, completely equal fragment size *S* (to be interpreted as if all the DNA was fragmented in *n* pieces with exactly the same length *S*) is


$$I=\frac{S-L+1}{S}\;\mathrm{if}\;S\geq L\;\mathrm{and}\;I=0\;\mathrm{if}\;S<L$$

This equation is visualized in Supplementary Figure S9. In practice, the DNA will never be fragmented in *n* pieces with exactly the same length *S*, but this mathematical equation allowed to understand differences in relative abundance if different species have different levels of fragment sizes concerning their DNA. Species with more fragmented DNA, such as *Lactococcus*, will tend to be less abundant in full-length 16S rRNA gene amplicon-based HTS compared with partial sequencing of the V4 region solely. Indeed, this was evident for Gouda cheese sample A1, for which both methods were applied at two different time points (Supplementary Figure S10).

### 4.3 Metagenome-assembled genome reconstruction using short-read metagenomic sequence reads

Using only the short-read HQ-MSR datasets, a total of 31 MAGs with at least 75% completion and no more than 10% redundancy were obtained for the five different HQ-MSR data subsets considered, encompassing 14 different species. Only one MAG of *Lc. cremoris* and not any MAG of *Lc. lactis* could be retrieved, despite their high relative abundance in all Gouda cheese samples examined. Since it was assumed that these high relative abundances might have hampered MAG recruitment, especially given the close similarity between *Lc. cremoris* and *Lc. lactis*, three new subsets, each containing HQ-MSR datasets of cheese samples related to one of the three starter culture mixtures applied and having low relative abundances for *Lactococcus,* were used to reconstruct *Lc. cremoris* and *Lc. lactis* MAGs, which did result in two *Lc*. *lactis* MAGs and one *Lc. cremoris* MAG. Additionally, two *T. halophilus* MAGs were obtained, using two subsets of the HQ-MSR datasets, related to the two distinct clusters of *T. halophilus* (Figure 4), and for which one MAG was obtained per cluster. The overall strategy to divide the 89 HQ-MSR datasets into distinct subsets resulted in a total of 56 MAGs related to 15 species (Supplementary Table S3).

### 4.4 Metagenome-assembled genome reconstruction using long- and short-read metagenomic sequence reads

The four Gouda cheese samples sequenced with both short- and long-read sequencing resulted in eight different co-assemblies (Supplementary Figure S3). The largest contigs as well as the highest contig N50 values were obtained with the co-assemblies obtained with the long-read HQ-MSRs solely (Supplementary Figure S11). The use of Flye with the parameter to keep haplotypes (COFH) did not result in a difference compared with the use of Flye without this parameter (COF). The three first iterations of Racon reduced the number of contigs from 16,721 in the COFH co-assembly to 12,611 in the COFHP one. This also came with a reduction of the total co-assembly size of 17.6%. The metaSPAdes co-assembly (COS) resulted in the highest number of contigs for all methods used. A reduction with 88.0% of the total number of contigs was obtained when metaMIC was applied (COS.MIC), while only reducing the total co-assembly size with 35%. Only six metagenomic bins could be reconstructed from the co-assemblies obtained with metaSPAdes and the short-read HQ-MSRs, of which five had a high completeness (Supplementary Table S4). The co-assemblies obtained with the long-read HQ-MSRs solely led to eight metagenomic bins with high completion. Using Flye with the parameter to keep haplotypes resulted in an unexpected reduction of the average bin completeness and a slight increase of the redundancy. The application of a polishing step using long and short reads resulted in a general improvement of the completeness and a reduction of the redundancy of the bins obtained, although the total number of reconstructed bins remained the same. In contrast, the application of metaMIC on the MEGAHIT co-assembly (COM.MIC) resulted in the appearance of an extra metagenomic bin, which resulted in a substantial reduction of the redundancy of the outlying bin from which it originated. Finally, Strainberry worsened the metagenomic binning in terms of completeness and redundancy, suggesting that the metagenome contained a microbial complexity that was difficult to handle with this tool. For example, the *T. halophilus* metagenomic bins had an average completeness and redundancy of 94.8 and 9.3%, respectively, for all long-read co-assemblies until Strainberry was applied, decreasing the completeness to 83.3% and increasing the redundancy to 32.5% (Supplementary Table S4). The metagenomic bins corresponding to *T. halophilus* obtained with the short-read co-assemblies were, in contrast, practically free of redundancy (<2.0%). The *Lc. laudensis* metagenomic bin also increased its redundancy from 0.9 to 25.6% upon the application of Strainberry.

Almost every metagenomic bin generated by any of the co-assembly methods used had a redundancy below 3%, suggesting the presence of one main population per bin (Supplementary Table S4). The exception, however, was a metagenomic bin identified as *Lc. cremoris*, whose redundancy was on average 89.6% for all unpolished co-assemblies (i.e., COF, COFH, COM, and COS) (Supplementary Table S4 and Supplementary Figure S12). This bin had an estimated genome size ranging from 4.18 to 6.86 Mbp, suggesting the presence of at least two populations of *Lc. cremoris*, or a closely related species. The polishing pipeline used to improve the long-read co-assembly (COFHP) resulted in 31.0% reduction of the redundancy of this particular bin, while improving the completeness with 0.86%. The COM.MIC co-assembly was the only one able to resolve this bin in two different ones, and it did so while maintaining high percentages of completeness for both of them. Furthermore, the genome size of the bins obtained (2.00 and 2.06 Mbp) were similar to the sizes expected for these LAB species (between 2.4 and 2.5 Mbp). One of these haplotype-resolved bins was identified as *Lc. lactis* instead, highlighting the importance of performing a successful haplotype phasing step.

The number of predicted gene functions was much higher for the MAGs obtained by COFH and COFHP than for those obtained by COM.MIC (Supplementary Table S5), mainly because they contained much more ‘hypothetical proteins’. Additionally, the long-read co-assemblies contained more predicted gene functions that were found at least twice within the MAGs. For each of the three co-assembly strategies applied, the MAGs had unique predicted gene functions that were not found with the two other strategies, and COFH and COFHP led to more predicted gene functions than COM.MIC (Supplementary Figure S13). COFHP yielded the most predicted gene functions for all MAGs, except for the *Lc. laudensis* and *Lacp. plantarum* MAGs, for which COFH yielded slightly more predicted gene functions compared with COFHP and considerably more compared with COM.MIC.

As an approximation to assess the correctness of the MAG assemblies, the MAGs were aligned to the 16S rRNA gene of the corresponding species for all eight co-assembly strategies applied. None of the MAGs reconstructed using short-read sequences solely contained a complete 16S rRNA gene, which was also the case for all other short-read-based MAGs obtained in the present study (Supplementary Table S3). In contrast, most MAGs reconstructed using long-read sequences solely resulted in complete 16S rRNA genes (Table 3), which also resulted in a correct copy number of these genes according to the rrnDB database in the case of *Lacc. paracasei*, *Leuc. pseudomesenteroides*, *Loil. rennini*, and *T. halophilus*. The COFHPB strategies showed also here to be less performant, with an incorrect 16S rRNA gene copy number for *Leuc. pseudomesenteroides*. All long-read-based MAGs also contained 5S and 23S rRNA genes, and all 20 tRNA genes (18 in the case of *W. thailandensis*). Aligning the 16S rRNA gene sequences from the MAGs with the ASVs obtained with full-length 16S rRNA amplicon-based HTS previously (Decadt et al., 2023), there was at least one co-assembly with exactly the same 16S rRNA gene sequence as in the ASVs, including the same ratio, for all species for which a correct copy number of 16S rRNA genes was found. This concerned five ASVs for *T. halophilus*, three different abundant ASVs for *Lacc. paracasei* and *Loil. rennini*, for both in a 3:1:1 ratio, and two ASVs for *Leuc. pseudomesenteroides* in a 3:1 ratio (Table 3). In the case of *T. halophilus*, for instance, the five 16S rRNA gene sequences retrieved with COFHP were identical to the corresponding ASVs, whereas those found with COF and COFH had only one gap compared with the corresponding ASVs, missing one thymine in a homopolymer sequence of seven thymines. Overall, COFHP retrieved the highest number of exact matches with the ASVs.

**Table 3:** Number of copies of the 16S rRNA gene in the metagenome-assembled genomes (MAGs) retrieved by long-read metagenomic sequencing (COF and COFH), and long-read metagenomic sequencing with polishing of the short reads (COFHP and COFHPB) of whole-community DNA from the Gouda cheese samples.

MAG	COF	COFH	COFHP	COFHPB	rrnDB
*Lacticaseibacillus paracasei*	**5** (1)	**5** (1)	**5** (5)	**5** (5)	5
*Lactiplantibacillus plantarum*	2 (1)	2 (1)	2 (2)	2 (2)	5
*Lactococcus laudensis*	1 (1) + 2 SF	1 (1) + 1 SF	1 (1) + 1 SF	[1 (0)]	Genus: 6
*Leuconostoc pseudomesenteroides*	**4** (4)	**4** (4)	**4** (3)	3 (1)	4
*Loigolactobacillus rennini*	**5** (1)	**5** (1)	**5** (5)	**5** (5)	Genus: 5
*Tetragenococcus halophilus*	**5** (0)	**5** (0)	**5** (5)	[**5** (1)]	5
*Weissella thailandensis*	1 (0) + 3 MF	1 (0) + 3 MF	1 (0) + 3 MF	1 (0) + 3 MF	8

Incomplete 16S rRNA genes are referred to as small fragments (SF, <100 bp) and medium fragments (MF, 450–550 bp). In the case the metagenomic bins did not meet the qualifications of a MAG, the number is indicated between squared brackets. Numbers underlined in bold are in accordance with the median copy number as found in the rrnDB (Stoddard et al., 2015) for the species, or the genus if no species data were available (last column). The number between brackets is the number of 16S rRNA gene sequences that had an exact match with an amplicon sequence variant (ASV) found in the corresponding samples previously (Decadt et al., 2024b).

### 4.5 Metagenome-assembled genome comparison and functional analysis

As multiple MAGs were obtained for the species *Lacc. paracasei* (8), *Lacp. plantarum* (3), *Lc. laudensis* (8), *Lc. cremoris* (2)*, Lc. lactis* (2)*, Leuc. pseudomesenteroides* (8), *Loil. rennini* (8), and *T. halophilus* (9) (Supplementary Table S3), those MAGs were compared per species based on full ANI percentage. Overall, small differences were found for all these species. In the case of *T. halophilus*, the MAGs of the two different subsets (T1 and T2) shared a full ANI value of only 77.5% and an ANI value of 97.8%.

A basic functional analysis was done for the MAGs with the highest completion per species (Supplementary Table S3) and for all MAGs obtained with COFHP. In the case of *Lc. cremoris* and *Lc. lactis*, all MAGs obtained and listed in Supplementary Table S3, as well as those retrieved by COM.MIC, were considered. For *T. halophilus*, the two MAGs representing both clusters mentioned above were used. The genes of interest were related to lactate production, diacetyl/acetoin production, and biogenic amine production, and also various aminopeptidases were targeted (Supplementary Table S6). Whereas the lactococci and *T. halophilus* contained only genes encoding enzymes involved in the production of l-lactate, *Leuc. pseudomesenteroides* contained only genes encoding enzymes involved in the production of d-lactate. The other LAB species contained genes involved in the production of both, of which only *Lacp. plantarum* also contained all genes involved in lactate racemisation. All species, except for *T. halophilus*, contained genes encoding acetolactate synthase and acetolactate decarboxylase involved in diacetyl/acetoin production. Genes related to the production of biogenic amines were mainly found in *Loil. rennini*, such as genes encoding an inducible ornithine decarboxylase producing putrescine, the related ornithine carbamoyltransferase and putrescine-ornithine antiporter, and a tryptophan decarboxylase. *Loigolactobacillus rennini* also contained genes encoding a glutamate decarboxylase and a glutamate/gamma-aminobutyrate antiporter.

In the case of the *T. halophilus* MAGs, no genes related to biogenic amine production were found, although high sequence identity (>99%) was obtained with the histidine decarboxylase-encoding genes *hdcA* and *hdcB*, and the histidine/histamine antiporter-encoding gene *hdcP* in the contigs of the three subsets related to starter culture mixtures A, B, and C before binning. For the subset related to the cheeses with crack defects, only a match with *hdcP* was found, whereas no matches were found for any of the three histamine-related genes in the subset corresponding with the samples from the longitudinal study of a Gouda cheese production batch, nor in the long-read subset. Specifically in the metagenomic DNA of the subset of samples corresponding with T1, not any of the three histamine-related genes were found, whereas all three histamine-related genes were found in the metagenomic DNA of the subset of samples corresponding with T2.

Whether most genes encoding aminopeptidases were found in all species (i.e., aminopeptidase C, aminopeptidase N, aminopeptidase YpdF, methionine aminopeptidase, neutral endopeptidase, and peptidase T), a gene encoding aminopeptidase E was not found in the lactococci and *Leuc. pseudomesenteroides* MAGs, whereas a gene encoding aminopeptidase PepS was only present in *Lacc. paracasei* and *T. halophilus*, and a gene encoding carboxypeptidase was only present in *Leuc. pseudomesenteroides* and *T. halophilus*. A gene encoding glutamyl endopeptidase, based on which glutamate can be released, was only found in *Loil. rennini*.

## 5 Discussion

Up to now, shotgun metagenomic sequencing has not been investigated to study the intraspecies diversity and functional potential of the Gouda cheese microbiota (Smid et al., 2014). The current study tackled the microbial composition of 89 Gouda cheese and cheese production-related samples using short-read shotgun metagenomics. It allowed to determine the high relative abundance of *Lc. cremoris* in the Gouda cheese starter cultures used and throughout the Gouda cheese production chain. Further, the bacterial identities were compared with data obtained previously by amplicon-based HTS of the full-length 16S rRNA gene, namely, for the whole Gouda cheese production chain (Decadt et al., 2024b), Gouda cheese wheels showing batch-to-batch variability in organoleptic quality (Decadt et al., 2023), and Gouda cheeses with crack defects (Decadt et al., 2024a). The short-read metagenomic data showed a higher relative abundance of *Lc. cremoris* and a lower one of NSLAB during cheese ripening, compared with the amplicon-based HTS. Additionally, these short-read metagenomic sequence data allowed to retrieve MAGs of 15 different species and, hence, perform a functional analysis to unravel the contribution of both SLAB and NSLAB to Gouda cheesemaking. Finally, long-read metagenomic sequencing of four Gouda cheese samples yielded an additional dataset that could be used for taxonomic analysis and MAG recruitment as well as functional analysis. Those MAGs were of a higher quality than those obtained by short-read metagenomic sequencing. Consequently, these data allowed to compare the short- and long-read metagenomic sequencing approaches.

For short-read metagenomics, the taxonomic identification at genus level was very similar for all five alignment-based methodologies followed. The NSLAB species *Loigolactobacillus* was an exception, as it was considered not abundantly present based on the methods relying on the RefSeq nucleotide database. Indeed, according to the phylogenetic tree of StrainPhlAn, the *Loigolactobacillus* species present in the Gouda cheese samples examined deviated from the reference strain. Hence, this illustrated the disadvantage of databases with only one representative genome per species. Further, the analysis relying on DIAMOND with the nr database showed a low relative abundance of viral DNA. In the case of the analysis with Kraken2, relying on the RefSeq nucleotide database, there was even not any hit with viral DNA entries. Instead, more hits with *Bacillus* entries occurred. Indeed, a *Skunavirus*-related sequence was present in a *B. thuringiensis* genome in this database, which is unusual given the specific association of *Skunavirus* with *Lactococcus*. Either the *Skunavirus* might yet be a prophage of *B. thuringiensis*, or it should be considered as a contaminating sequence. The presence of contaminating sequences in databases can never be excluded, and the chance that they are discovered increases with increasing taxonomic differences, which can be compared with the redundancy of yeast genomes with bacterial sequences (Donovan et al., 2018; Lupo et al., 2021).

Overall, Kraken2 applied with the nt database was the best approach to assign short-read metagenomic sequences, as it could identify both viruses and *Loigolactobacillus*, and it had the lowest percentage of unidentified reads. However, new tools are constantly developed and, although Kraken2 is a very fast method, other tools, such as KMCP, claim to offer a more accurate taxonomic profiling reliable at species level (Shen et al., 2023). However, tools as KMCP do not easily allow the detection of new species, unlike metagenomic recruitment plotting. The latter methodology was used in the present study and can give a good indication of the presence of new species, or species for which there is no genome sequence available yet in the public domain (Verce et al., 2019).

It was presumed that yeasts were not abundant in the samples investigated (Decadt et al., 2023, 2024b), which was confirmed by the genus-level identification of the current study, as yeasts had a relative abundance of less than 0.1% in all Gouda cheese samples examined. In general, the yeast counts of Gouda cheeses are between 1.0 and 3.0 log (CFU/g) (Aljewicz and Cichosz, 2017; Nalepa et al., 2020). Further, a meta-analysis of 184 various Irish artisan cheese samples has reported a mean relative abundance of 2% eukaryotic DNA, but a major presence (20%) of viral DNA (Walsh et al., 2020). The percentage of viral reads was tenfold lower in the Gouda cheese samples of the current study, but in contrast to other studies, the present study considered the age of the cheeses as well as the location of the samples in the cheeses, which revealed a significantly higher viral abundance in long-ripened cheeses and in the rinds. The lower water activity in the rinds and long-ripened Gouda cheeses, combined with a higher relative abundance of NSLAB (Decadt et al., 2023, 2024b), might generate bacterial stress on *Lactococcus*, and may further induce the lytic cycle of its *Skunavirus* prophage (Garneau and Moineau, 2011), resulting in the multiplication of the phage and, hence, a higher relative abundance of viral DNA.

At species level, short-read shotgun metagenomics confirmed the presence of the Gouda cheese bacterial species that have been reported using amplicon-based HTS targeting the full-length 16S rRNA gene previously (Decadt et al., 2023, 2024a, 2024b). Another study comparing short-read shotgun metagenomics with amplicon-based HTS of the full-length 16S rRNA gene (applying the ONT platform) has shown that the use of both methods lead to the same dairy core microbiota, but that minorities might differ, whereas amplicon-based HTS detects fewer species (Rubiola et al., 2022). Compared to the latter study that has reported 13 core families and 1,078 different genera across all milk filter samples examined, the results of both methods were more similar in the present Gouda cheese study, which thus showed a more limited diversity of the abundant species (>0.4%). The main difference between both methods in the present study was the relative abundance of *Lc. cremoris*, which was significantly higher according to the short-read shotgun metagenomics data. Consequently, the relative abundance of the NSLAB was significantly lower according to short-read shotgun metagenomics compared with the amplicon-based full-length 16S rRNA gene HTS. The high relative abundance of *Lactococcus* was in line with the genus-level identification done with the five different alignment-based approaches applied. Whereas the data of the amplicon-based HTS are biased by the gene copy number, shotgun metagenomic data are biased by the genome size (Stothart et al., 2023). The median 16S rRNA gene copy number of *Lactococcus* is six, whereas that of *Tetragenococcus*, *Loigolactobacillus*, *Lactiplantibacillus*, and *Lacticaseibacillus* is five, and that of *Leuconostoc* four (Stoddard et al., 2015). This would suggest a higher relative abundance of *Lactococcus via* amplicon-based HTS compared with shotgun metagenomics, the opposite of what was found in the current study. The genome size of *Lc. cremoris* KW2 and that of *Lc. lactis* LAC460 is 2.43 Mbp, which is comparable with the genome size of *T. halophilus* MJ4 (2.39 Mbp), somewhat longer than that of *Lc. laudensis* DSM 28961, *Leuc. pseudomesenteroides* FDAARGOS_1003, and *Loil. rennini* DSM 20253 (2.30, 2.11, and 2.27 Mbp, respectively), and shorter than that of *Lacc. paracasei* 362.5013889 and *Lacp. plantarum* SRCM100442 (3.03 and 3.22 Mbp, respectively). Hence, the genome size differences can neither explain the discrepancy between amplicon-based HTS and short-read shotgun metagenomics. However, the additional long-read metagenomic sequencing of four Gouda cheese samples showed that the relative abundance of the species involved varied as a function of the read length, with a lower relative abundance of *Lc. cremoris* when considering a higher minimal read length cut-off. This indicated that the DNA of *Lc. cremoris* was more fragmented compared to that of *Lacc. paracasei*, *Leuc. pseudomesenteroides*, *Loil. rennini*, and *T. halophilus*, and thus allowed to explain the differences between amplicon-based HTS and short-read metagenomic sequencing. Indeed, for full-length 16S rRNA gene amplicon-based HTS, the 1,500-bp long gene needs to be complete (not fragmented) to be amplified. In contrast, the fragments sequenced during short-read metagenomic sequencing were only between 500 and 1,500 bp long, of which 2 × 250 bases were sequenced given the paired-end way of sequencing. Consequently, full-length 16S rRNA gene amplicon-based HTS will preferentially target species with a higher degree of DNA integrity compared with short-read metagenomic sequencing. This is also a main, and overlooked, difference between high-throughput amplicon-based HTS of the partial (for instance, the V4 hypervariable region of the 16S rRNA gene) *versus* the full-length 16S rRNA gene.

Knowing the underlying reason why the outcome of short-read metagenomic sequencing differed from that of full-length 16S rRNA gene amplicon-based HTS, did not answer the question which method would be the most accurate one. One could argue that the relative abundances of short-read metagenomic sequencing were the most reliable, since all DNA was directly sequenced, without the possible introduction of a PCR bias owing to eventual preferential amplifications. Indeed, the latter can be the case with amplicon-based HTS. However, the finding that differences in DNA fragment sizes were species-specific raised the question if these differences had a biological meaning. During cheese ripening, most *Lactococcus* cells go into a viable but not culturable state, or die (Gobbetti et al., 2018), which might be related to the higher fragmentation of the DNA of *Lactococcus* compared with other abundant species. If lactococci die in the same way as streptococci, for which DNA is fragmented before the cells lyse (Regev-Yochay et al., 2007), the short DNA fragments could come from degraded DNA of dead cells that were intact enough to end up in a cell pellet. Additionally, partially fragmented DNA can be present in extracellular vesicles that might be retained during cell pelleting (Bose et al., 2021; Yu et al., 2018). Consequently, DNAses can be added after cell pelleting to ensure the removal of extracellular DNA (Gatti et al., 2008), although it is more common to treat samples with propidium monoazide (PMA) that binds to extracellular DNA and DNA of membrane-compromised cells to do so (Emerson et al., 2017). Indeed, photoactivation degrades bound DNA, and only DNA from intact cells is then sequenced (Emerson et al., 2017). In cheese studies, significantly lower relative abundances of *Lactococcus* have been found after a PMA treatment (Barzideh et al., 2022; Porcellato and Skeie, 2016), which is comparable with the differences between shotgun metagenomics and full-length 16S rRNA gene amplicon-based HTS in the current study. Hence, there is an indication that the relative abundances obtained by full-length 16S rRNA gene amplicon-based HTS might reflect better the intact cell population compared with shotgun metagenomics. However, more research should be performed, including PMA treatments and/or the use of DNAses, to confirm this hypothesis.

The cheese microbiota forms an ecosystem that has a rather limited microbial species diversity but contains different strains of some of those species (Smid et al., 2014). The short-read and long-read metagenomic datasets obtained in the current study served as ideal datasets to assess various bioinformatics tools and strategies as to their capabilities to be able to distinguish those strains. Based on StrainPhlAn, two main intraspecies clusters could be distinguished for both *Leuc. pseudomesenteroides* and *T. halophilus*, which was completely in line with the ASV-based clustering reported previously (Decadt et al., 2023). It indicated the presence of (at least) two strains for each species. Whereas StrainPhlAn was applied to assess whether it could distinguish the different strains present for some of these species, it was not able to do so, as it only considered one consensus cluster, making this tool unsuited to analyze metagenomes in which multiple strains of the same species are to be expected. Likewise, full-length 16S rRNA gene amplicon-based HTS might not always be able to distinguish different strains, as the 16S rRNA gene can be very conserved in some species, such as staphylococci (Van Reckem et al., 2020).

With regard to metagenomic recruitment plotting, this approach allowed to pinpoint the most likely subspecies in the case of the SLAB *Lc. cremoris* and *Lc. lactis*, and the NSLAB *Lacc. paracasei* and *Leuc. mesenteroides*. It could not assign the *T. halophilus* reads to one subspecies, possibly because the difference between the reference genomes of both subspecies of this species is as small as between any *T. halophilus* genome. However, metagenomic recruitment plotting indicated that the HQ-MSRs that matched with *Weissella* genomes did so for *W. jogaejeotgali* but not for *W. thailandensis*. This suggested that *W. jogaejeotgali* might be considered as a subspecies of *W. thailandensis*, and not just as a synonym (Kwak et al., 2019). Although MAGs could be constructed for a total of 15 species, there was never more than one MAG per species within any subset of HQ-MSR data, despite the strain diversity within some species. The poor MAG retrieval for *Lc. lactis* and *Lc. cremoris* could be improved by using less cheese samples with a lower relative abundance of both lactococci. A knowledgeable selection of the samples is thus very important to obtain reliable results, as a high relative abundance of a species might at some point be counterproductive in generating MAGs of that species if multiple strains are present. Similarly, using more samples might increase redundancy rather than completion. Likewise, only when carefully selecting and combining the HQ-MSR datasets that already showed to be related to one of the *T. halophilus* clusters, two distinct MAGs for the two main *T. halophilus* clusters could be obtained. However, not all differences between the two *T. halophilus* clusters were included in the corresponding MAGs, since plasmid-encoded genes are mostly not incorporated into MAGs (Vermote et al., 2023). In particular, genes related to histamine production by *T. halophilus* are plasmid-encoded and this plasmid is not present in all strains (Ma et al., 2024). Indeed, the HQ-MSR datasets related to one *T. halophilus* cluster contained all genes associated with histamine production, whereas the HQ-MSR datasets related to the other *T. halophilus* cluster did not contain any of those genes. Hence, when different MAGs of the same species are obtained, it might be a good practice to also consider the genes in the contigs that are not part of the MAGs to detect missed functionalities.

According to the minimum information about a metagenome-assembled genome (MIMAG) standards proposed by the Genomic Standards Consortium, MAGs need a completion of >90%, a redundancy of <5%, and the presence of all 23S, 16S, and 5S rRNA genes, and at least 18 tRNA genes, to be qualified as high-quality MAGs (Bowers et al., 2017). Whereas several short-read MAGs in the present analysis met these requirements for completion and redundancy, none of them contained the requested rRNA and tRNA genes. Hence, it seemed that MAGs obtained by short-read metagenomic sequencing cannot meet these criteria, at least in the current study. Long-read metagenomic sequencing might be necessary to obtain high-quality MAGs. Indeed, all seven long-read sequencing MAGs of the present study contained all the genes requested, and only the MAG of *W. thailandensis*, with a completion lower than 90%, did not meet all those requirements to be considered as a high-quality MAG.

The retrieval of 16S rRNA genes from MAGs generated using short-read metagenomic sequencing is known to be problematic (Yuan et al., 2015). Only 7% of the MAGs obtained from more than 3,500 human gut metagenomes contained 16S rRNA genes, with a significantly lower copy number compared to complete RefSeq genomes (Hiseni et al., 2022). Although there are tools available to optimize 16S rRNA gene retrieval from MAGs obtained by short-read metagenomic sequencing (Pericard et al., 2018; Song et al., 2022), they could not be applied to optimize the correct retrieval of other genes witnessing the same problem because of a high similarity between different MAGs. In addition, the cheese microbiota has a significant level of horizontal gene transfer (Bonham et al., 2017; Walsh et al., 2020), and it is, therefore, questionable if short-read metagenomic sequencing can always deal with this.

In addition to the superior retrieval of the different rRNA genes, the long-read shotgun metagenomic sequencing, applied for four Gouda cheese samples, yielded more MAGs, compared with short-read shotgun metagenomic sequencing. Also, these MAGs contained more predicted gene functions. This suggested that long-read metagenomics might be superior to short-read metagenomics with regard to MAG retrieval, albeit that polishing the long-read sequencing data with short-read sequencing data further increased their accuracy. The latter was seen in the case of the 16S rRNA genes. Indeed, other studies have found a higher number of MAGs with ONT long-read sequencing compared to Illumina short-read sequencing (Meslier et al., 2022; Sereika et al., 2022). Further, although Illumina polishing significantly increases the MAG retrieval and quality in the case the R9.4.6 flow cell is used (Overholt et al., 2020; Sereika et al., 2022), long-read sequencing can already be used as stand-alone technique for (meta)genome sequencing (Sereika et al., 2022; Zhao et al., 2023) when the R10.4.1 flow cell is used.

The application of the Strainberry tool had a detrimental effect on the quality of the MAGs. Moreover, it failed completely to separate strains. In the case of *Lc. cremoris* and *Lc. lactis*, a large intraspecies variety, combined with a high similarity between both species, might be the main cause of this failure (Smid et al., 2014). Although Strainberry has difficulties with separating very closely related strains (Vicedomini et al., 2021), it can be assumed that such separation is difficult for all tools. Hence, strain-level resolution of undefined, mesophilic starter cultures that typically contain several lineages (Erkus et al., 2013; Smid et al., 2014), is probably not feasible yet by the current metagenomic approaches. More philosophically, all MAGs retrieved were in fact a consensus of genomes that are very closely related and might or might not be the same strain. Moreover, there is no clear cut-off concerning similarity between two genomes to be considered as belonging to the same strain (Van Rossum et al., 2020), suggesting that strain-level metagenomics is more an ultimate aim than a real possibility. This does not exclude the possibility to detect different clusters within a species, as shown in the case of *T. halophilus* during the present study. Of the two main *T. halophilus* clusters, only cluster 2 contained genes for histamine production, corroborating with correlations of metabolites and the corresponding ASVs (Decadt et al., 2023). This illustrates the usefulness of intraspecies metagenomics. The fact that these findings come from a selection of specific Gouda cheese samples containing only one *T. halophilus* cluster illustrates an alternative, less black-box approach compared with the use of so-called strain-specific tools that did not give satisfying results.

The functional analysis of the MAGs of all species mentioned above confirmed L-lactate production by lactococci and *T. halophilus* (Kim, 2014; Chun et al., 2019), diacetyl/acetoin production by all species except *T. halophilus* (Chun et al., 2019; McAuliffe et al., 2019), aminopeptidase activity by all species (Savijoki et al., 2006; Kieliszek et al., 2021), and biogenic amine production by *Loil. rennini* and *T. halophilus* (Kazou et al., 2017; Decadt et al., 2024a; Ma et al., 2024).

## 6 Conclusion

Metagenomic sequencing gave a deep insight into the composition of the Gouda cheese microbiota. The SLAB *Lc. cremoris* was the most abundant species in all Gouda cheeses examined, thereby occurring from the starter addition upon, including the cores and rinds of the long-ripened cheeses, followed by the SLAB *Lc. lactis*. Whereas around 2% of the DNA in the Gouda cheeses originated from phages, the yeast fraction was negligible. The relative abundances of NSLAB species as found by metagenomic sequencing was significantly lower compared with those obtained by full-length 16S rRNA gene amplicon-based HTS. The identities of all species found in each sample were the same for both sequencing methodologies, even on intraspecies level. Long-read metagenomic sequencing showed a species-specific fragmentation of the DNA, with lactococcal DNA being more fragmented compared with that of the SLAB *Leuc. pseudomesenteroides* and the NSLAB *Lacc. paracasei*, *Loil. rennini*, and *T. halophilus*. It is likely that a higher degree of DNA fragmentation was related to a higher degree of inactivity or death of the corresponding species. Hence, full-length 16S rRNA gene amplicon-based HTS might give a more accurate view on the relative abundances of the active bacteria compared with both short-read metagenomic sequencing and partial 16S rRNA gene amplicon-based HTS, and might be a good alternative to PMA treatments or other approaches to separate the active from the inactive and dead microbial cells. Additionally, short-read metagenomic sequencing could not result in high-quality MAGs, but long-read metagenomics could. The latter might thus be the better choice for MAG retrieval and subsequent functional analysis. However, both short-read and long-read metagenomic sequencing had difficulties with obtaining MAGs for *Lc. cremoris* and *Lc. lactis*, owing to their high level of intraspecies diversity. The use of less samples, with low relative abundances of these species, was the best approach to obtain lactococcal MAGs, suggesting that more data do not always lead to better results. Although intraspecies differences were found, for instance, for *T. halophilus*, no metagenomic approach could really give strain-level insights. A functional analysis of the MAGs of all species mentioned above revealed genes related to l-lactate production (lactococci and *T. halophilus*), d-lactate production (*Leuc. pseudomesenteroides*), diacetyl/acetoin production (not *T. halophilus*), aminopeptidase activity (all species), and biogenic amine production (*Loil. rennini* and *T. halophilus*), confirming earlier metabolomic data.

## Data Availability

The datasets presented in this study can be found in online repositories. The names of the repository/repositories and accession number(s) can be found: https://www.ebi.ac.uk/ena, PRJEB77845.
