# Supplementary material for: Long-read metagenomics gives a more accurate insight into the microbiota of long-ripened gouda cheeses
Source: Front Microbiol. 2025 Mar 24;16:1543079. doi: 10.3389/fmicb.2025.1543079 (PMC11973332; doi:10.3389/fmicb.2025.1543079)
Supplement: Supplementary file 1 [file Data_Sheet_1.pdf]

Supplementary Material

Long-read metagenomics gives a more accurate insight into the microbiota of long-ripened Gouda cheeses

Hannes Decadt, Cristian Díaz-Muñoz, Louise Vermote, Inés Pradal, Luc De Vuyst, and Stefan Weckx*

Research Group of Industrial Microbiology and Food Biotechnology (IMDO), Faculty of Sciences and Bioengineering Sciences, Vrije Universiteit Brussel, Brussels, Belgium

*** Correspondence:** Stefan Weckx, Stefan.weckx@vub.be

Supplementary Tables

Table S1. Relative abundance of viruses based on short-read metagenomic sequencing for the 23 Gouda cheeses with batch-to-batch variability and the cheeses with crack defects (names as explained in Table 1) and the processed milk and cheese samples from the longitudinal study of a Gouda cheese production batch (C, curd; W, whey), using Kraken2 [NCBI nucleotide database (NCBI nt)].

	Age (weeks)			Zone		Time	Milk/	
Cheese	36	75	Cheese	Cr	Z	point	Core	Rind
A1	0.73		M1a	0.39	0.25	0.0 h	0.01	
A2	3.41	7.39	M1b	0.35	0.24	0.9 h	1.68	
A3	0.73		M1c	0.50	0.39	1.8 h C	1.17	
A4	2.03		M2	0.28	0.25	1.8 h W	0.77	
A5	0.51		L1a	0.68	0.49	3.7 h	1.28	
A6	1.00		L1b	0.52	0.70	7.5 h	0.29	
A7	0.62	0.82	L1c	0.63	0.53	3 d	0.33	
A8	0.99	1.64	L2	0.41	0.57	2 w	0.66	
B1	1.68	1.56	L3	0.67	0.71	4 w	1.32	2.99
B2	1.31		XL	0.47		8 w	0.34	0.45
B3	0.55					12 w	1.40	3.27
B4	2.07					18 w	1.07	1.51
B5	0.47	0.38				25 w	0.71	5.60
B6	0.57	0.55				31 w	1.81	4.94
B7	1.16					36 w	2.16	4.79
C1	0.66					40 w	1.95	2.46
C2	0.28					45 w	3.70	7.72
C3	0.77	8.08				55 w	3.66	7.93
C4	0.31	0.55				65 w	3.10	6.61
C5	0.46					75 w	7.83	14.77
C6	0.10					85 w	2.20	3.31
C7	0.14	0.21				100 w	4.13	3.29

C8	0.16	0.91

Table S2. The ratio of reads obtained through metagenomic recruitment plotting between two (sub)species, expressed as average values ± standard deviations over all samples examined.

Subspecies that were compared	Ratio
Lacticaseibacillus paracasei subsp. *tolerans* to *Lacc. paracasei* subsp. *paracasei*	0.55 ± 0.14
Lactococcus cremoris subsp. *tructae to Lc. cremoris* subsp. *cremoris*	0.18 ± 0.02
Lactococcus lactis subsp. *hordniae* to *Lc. lactis* subsp. *Lactis*	0.22 ± 0.02
Leuconostoc falkenbergense to *Leuconostoc pseudomesenteroides*	0.91 ± 0.08
Leuconostoc mesenteroides subsp. *dextranicum* to *Leuc. mesenteroides* subsp. *cremoris*	0.16 ± 0.06
Leuconostoc mesenteroides subsp. *jonggajibkimchii* to *Leuc. mesenteroides* subsp. *cremoris*	0.12 ± 0.07
Leuconostoc mesenteroides subsp. *mesenteroides* to *Leuc. mesenteroides* subsp. *cremoris*	0.04 ± 0.02
Leuconostoc mesenteroides subsp. *sake** to *Leuc. mesenteroides* subsp. *cremoris*	0.09 ± 0.09
Tetragenococcus halophilus subsp. *flandriensis* to *T. halophilus* subsp. *halophilus*	0.83 ± 0.14
*Weissella thailandensis to Weissella jogaejeotgali**	0.19 ± 0.08

*, currently not a valid (sub)species

Table S3. Metagenome-assembled genomes (MAGs, > 75 % completion and < 10 % redundancy) derived from the ten different subsets of the 89 high-quality metagenomic sequence read datasets obtained through short-read metagenomic sequencing of whole-community DNA from the Gouda cheese samples. The MAGs with an asterisk (*) were further used for functional analysis.

**Species for which a MAG was retrieved**	**Completion (%)**	**Redundancy (%)**	**Size (Mbp)**
Longitudinal study			
Brachybacterium alimentarium	87.3	5.6	3.238
Brevibacterium aurantiacum	76.1	2.8	2.974
Chryseobacterium haifense	78.9	2.8	1.572
Corynebacterium variabile	95.8	2.8	3.029
Lactococcus laudensis	77.5	2.8	1.309
Leuconostoc pseudomesenteroides	94.4	1.4	1.841
Staphylococcus equorum	100.0	0.0	2.555
Tetragenococcus halophilus	97.2	1.4	2.111
Gouda cheeses with crack defects			
Lacticaseibacillus paracasei	97.2	1.4	2.319
Lactococcus laudensis	93.0	4.2	1.922
Loigolactobacillus rennini	87.3	2.8	1.874
Tetragenococcus halophilus	85.9	5.6	2.450
Weissella thailandensis	80.3	2.8	1.595
Gouda cheeses with batch-to-batch variability, starter culture mixture A			
Lacticaseibacillus paracasei	98.6	0.0	2.847
*Lactococcus laudensis**	98.6	4.2	2.812
Leuconostoc pseudomesenteroides	98.6	2.8	1.955
Loigolactobacillus rennini	93.0	0.0	2.030
Propionibacterium freudenreichii	94.4	1.4	2.441
Tetragenococcus halophilus	94.4	5.6	2.892
Gouda cheeses with batch-to-batch variability, starter culture mixture B			
Lacticaseibacillus paracasei	98.6	0.0	2.538
*Lactiplantibacillus plantarum**	100.0	1.4	2.833
Lactococcus laudensis	77.5	4.2	2.002
Leuconostoc pseudomesenteroides	88.7	5.6	1.689
*Loigolactobacillus rennini**	98.6	0.0	2.098
Gouda cheeses with batch-to-batch variability, starter culture mixture C			

Lacticaseibacillus paracasei	98.6	0.0	2.703
Lactiplantibacillus plantarum	77.5	1.4	2.891
*Lactococcus cremoris**	81.7	2.8	2.207
Lactococcus laudensis	97.2	4.2	2.782
Leuconostoc pseudomesenteroides	88.7	4.2	1.774
Loigolactobacillus rennini	94.4	2.8	1.863
Tetragenococcus halophilus	97.2	2.8	2.381
Cheese cores with low relative abundance of *Lactococcus* from starter culture mixture A			
Lacticaseibacillus paracasei	98.6	2.8	2.499
*Lactococcus lactis**	85.9	7.0	2.315
Lactococcus laudensis	91.5	4.2	2.072
*Leuconostoc pseudomesenteroides**	100.0	1.4	1.743
Loigolactobacillus rennini	98.6	2.8	2.112
Tetragenococcus halophilus	94.4	2.8	2.353
Cheese cores with low relative abundance of *Lactococcus* from starter culture mixture B			
Lacticaseibacillus paracasei	98.6	0.0	2.423
Loigolactobacillus rennini	95.8	0.0	2.223
Tetragenococcus halophilus	98.6	5.6	2.403
Cheese cores with low relative abundance of *Lactococcus* from starter culture mixture C			
Lacticaseibacillus paracasei	98.6	1.4	2.696
*Lactococcus lactis**	80.3	7.0	2.036
Lactococcus laudensis	93.0	4.2	1.777
Leuconostoc pseudomesenteroides	100.0	1.4	1.695
Loigolactobacillus rennini	94.4	1.4	1.865
Tetragenococcus halophilus	98.6	2.8	2.151
Tetragenococcus halophilus cluster 1			
*Lactococcus cremoris**	87.3	5.6	2.079
Lactococcus laudensis	93.0	7.0	1.904
Leuconostoc pseudomesenteroides	100.0	2.8	1.711
Loigolactobacillus rennini	98.6	4.2	2.305
*Tetragenococcus halophilus**	98.6	4.2	2.397
Tetragenococcus halophilus cluster 2			
*Lacticaseibacillus paracasei**	98.6	0.0	2.445
Lactiplantibacillus plantarum	84.5	1.4	2.654
Lactococcus laudensis	95.8	5.6	1.954
Leuconostoc pseudomesenteroides	78.9	2.8	1.693
*Tetragenococcus halophilus**	98.6	1.4	2.427

Table S4. Metagenomic bins (> 10 % of completeness) for the whole-community DNA samples of the longitudinal study of a Gouda cheese production batch that were sequenced by both short- and long-read sequencing. The co-assembly code of the eight methods applied is as explained in the legend of Figure S3.

**Species**	**Completion (%)**	**Redundancy (%)**	**Size (Mbp)**	**Method**
Cellulosimicrobium	31.7	0.2	3.070	COF
Cellulosimicrobium	30.6	0.2	2.998	COFH
Cellulosimicrobium	36.1	0.0	3.044	COFHP
Cellulosimicrobium	36.1	0.0	3.042	COFHPB
Chryseobacterium	45.8	1.4	1.833	COF
Chryseobacterium	44.8	1.4	1.830	COFH
Chryseobacterium	45.8	0.7	1.830	COFHP
Chryseobacterium	46.3	1.2	1.829	COFHPB
Enterococcus / Streptococcus	82.5	11.4	2.064	COF
Enterococcus / Streptococcus	80.7	9.6	2.074	COFH
Enterococcus / Streptococcus	83.6	13.2	1.990	COFHP
Enterococcus / Streptococcus	65.2	9.7	1.564	COFHPB
Enterococcus / Streptococcus	11.7	0.0	0.638	COM
Enterococcus / Streptococcus	11.7	0.0	0.638	COM.MIC
Lacticaseibacillus paracasei	95.6	0.7	2.991	COF
Lacticaseibacillus paracasei	95.6	0.0	2.945	COFH
Lacticaseibacillus paracasei	95.6	0.0	2.925	COFHP
Lacticaseibacillus paracasei	95.6	0.0	2.908	COFHPB
Lacticaseibacillus paracasei	51.5	1.0	1.503	COM
Lacticaseibacillus paracasei	51.3	0.8	1.483	COM.MIC
Lacticaseibacillus paracasei	40.9	0.8	1.097	COS
Lacticaseibacillus paracasei	40.9	0.8	1.085	COS.MIC
Lactiplantibacillus plantarum	94.2	2.6	3.086	COF
Lactiplantibacillus plantarum	94.2	2.6	3.180	COFH
Lactiplantibacillus plantarum	96.0	2.8	3.077	COFHP
Lactiplantibacillus plantarum	93.3	2.8	3.050	COFHPB
Lactiplantibacillus plantarum	10.6	0.0	0.643	COM
Lactiplantibacillus plantarum	10.8	0.0	0.816	COM.MIC
Lactococcus cremoris (1)	95.7	87.0	6.856	COF
Lactococcus cremoris (1)	95.7	99.0	6.172	COFH
Lactococcus cremoris (1)	96.6	68.0	4.388	COFHP
Lactococcus cremoris (1)	91.4	123.2	6.483	COFHPB
Lactococcus cremoris (1)	100.0	84.9	4.436	COM
Lactococcus cremoris (1)	94.0	8.2	2.153	COM.MIC
Lactococcus cremoris (1)	98.1	87.6	4.177	COS
Lactococcus cremoris (1)	98.1	87.6	4.132	COS.MIC
Lactococcus cremoris (2)	39.0	15.8	4.080	COF
Lactococcus cremoris (2)	43.6	15.7	3.446	COFH
Lactococcus cremoris (2)	18.5	6.9	4.857	COFHP
Lactococcus cremoris (2)	25.9	4.3	5.483	COFHPB
Lactococcus cremoris (2)	15.0	2.9	3.556	COM

Lactococcus cremoris (2)	16.2	3.1	3.704	COM.MIC
Lactococcus lactis	83.0	2.9	2.059	COM.MIC
Lactococcus laudensis	97.4	4.5	2.506	COF
Lactococcus laudensis	97.7	2.3	2.269	COFH
Lactococcus laudensis	96.6	0.9	2.180	COFHP
Lactococcus laudensis	81.3	25.6	2.300	COFHPB
Lactococcus laudensis	93.9	1.8	2.040	COM
Lactococcus laudensis	93.6	0.8	1.996	COM.MIC
Lactococcus laudensis	95.9	0.5	1.998	COS
Lactococcus laudensis	96.2	0.5	2.002	COS.MIC
Leuconostoc mesenteroides	38.2	0.0	1.154	COF
Leuconostoc mesenteroides	37.4	0.9	1.427	COFH
Leuconostoc mesenteroides	34.6	0.0	1.653	COFHP
Leuconostoc mesenteroides	39.7	1.7	1.364	COFHPB
Leuconostoc pseudomesenteroides	97.4	0.2	2.090	COF
Leuconostoc pseudomesenteroides	97.4	0.2	2.092	COFH
Leuconostoc pseudomesenteroides	100.0	0.2	2.093	COFHP
Leuconostoc pseudomesenteroides	88.9	1.5	2.134	COFHPB
Leuconostoc pseudomesenteroides	97.9	1.2	1.866	COM
Leuconostoc pseudomesenteroides	100.0	0.2	1.888	COM.MIC
Leuconostoc pseudomesenteroides	100.0	0.2	1.937	COS
Leuconostoc pseudomesenteroides	100.0	0.2	1.938	COS.MIC
Loigolactobacillus rennini	98.0	1.1	2.357	COF
Loigolactobacillus rennini	98.0	1.1	2.354	COFH
Loigolactobacillus rennini	99.5	1.1	2.373	COFHP
Loigolactobacillus rennini	99.5	1.1	2.320	COFHPB
Loigolactobacillus rennini	99.2	1.1	2.026	COM
Loigolactobacillus rennini	99.2	1.1	2.036	COM.MIC
Loigolactobacillus rennini	99.2	1.1	2.024	COS
Loigolactobacillus rennini	99.2	1.1	2.016	COS.MIC
Tetragenococcus halophilus	93.3	9.3	3.219	COF
Tetragenococcus halophilus	92.6	9.9	3.270	COFH
Tetragenococcus halophilus	98.7	8.6	3.246	COFHP
Tetragenococcus halophilus	83.3	32.5	3.600	COFHPB
Tetragenococcus halophilus	98.7	0.0	2.678	COM
Tetragenococcus halophilus	98.7	0.0	2.634	COM.MIC
Tetragenococcus halophilus	99.3	2.0	2.661	COS
Tetragenococcus halophilus	99.3	2.0	2.693	COS.MIC
Weissella thailandensis	81.1	2.2	2.334	COF
Weissella thailandensis	79.5	2.2	2.035	COFH
Weissella thailandensis	83.0	1.5	2.141	COFHP
Weissella thailandensis	79.6	2.5	2.215	COFHPB

Table S5. Number of predicted genes for the MAGs in whole-community DNA samples of the longitudinal study of a Gouda cheese production batch sequenced by both short- and long-read sequencing. The best methods using long reads only (COFH), using long reads with polishing by short reads (COFHP), and using short reads only (COM.MIC) were used; the co-assembly code is as explained in the legend of Figure S3. A, number of all predicted genes; F, number of predicted genes to which a function could be assigned; U, number of unique predicted genes to which a function could be assigned. The metagenomic bins obtained by COM.MIC of *Lacticaseibacillus paracasei* and *Lactiplantibacillus plantarum* did not qualify as MAGs (> 75 % completion and < 10 % redundancy; Table S4) but were used for comparison purposes.

**Species**	**COFH**			**COFHP**			**COM.MIC**		
	A	F	U	A	F	U	A	F	U
Lacticaseibacillus paracasei	3323	1610	1135	3191	1592	1156	1520	585	513
Lactiplantibacillus plantarum	3684	1713	1205	3359	1620	1196	866	251	219
Lactococcus laudensis	2475	1302	1057	2189	1216	1045	1951	1083	955
Leuconostoc pseudomesenteroides	2502	1247	950	2172	1189	966	1951	1036	925
Loigolactobacillus rennini	2734	1556	1079	2277	1448	1089	1973	1203	1034
Tetragenococcus halophilus	4299	2069	1183	3185	1830	1195	2622	1453	1175
Weissella thailandensis	3223	1250	861	3262	1269	889			

Table S6. Overview of gene functions found in the metagenome-assembled genomes (MAGs) of the different species identified through short-read and long-read metagenomic sequencing of Gouda cheese samples. For each species, the MAGs indicated in Supplementary Table S3 were considered, and all MAGs obtained with COFHP (and COM.MIC for *Lc. cremoris* and *Lc. lactis*) were used as well. Green (+), genes present in all MAGs of that species investigated; yellow (s), genes present in at least one MAG investigated; white, genes not found in any MAG investigated.

	Laticaseibacillus paracasei	*Laciplantibacillus plantarum*	*Lactococcus cremoris*	*Lactococcus lactis*	*Lactococcus laudensis*	*Leuconostoc pseudomesenteroides*	*Loigolactobacillus rennini*	*Tetragenococcus halophilus*
L-lactate dehydrogenase (*ldh*)	+	+	+	+	+		+	s
D-lactate dehydrogenase (*ldh*)	+	+				+	+	
Quinone-dependent D-lactate dehydrogenase (*dld)*			s			+		
Lactate racemase and accessory genes (*larABCDE* and *larMNQO)*		s						
Lactate racemase regulatory gene (*larR)*		s	+	+				
Acetolactate synthase (*alsS)*	+	+	s	s	+	+	+	
Acetolactate synthase (*ilvB* and *ilvH)*		+	s	+	+			
Alpha-acetolactate decarboxylase (*aldB*)	+							
Alpha-acetolactate decarboxylase (*aldC*)		+	+	+	+	+	+	+
Diacetyl reductase [(S)-acetoin formation] (*budC*)	s		+	+			+	
Lactate utilization protein A, B, and C (*lutA*, *lutB*, and *lutC*)	+	+	+	+			+	
Ornithine carbamoyltransferase (*arcB*)		s	+	+	+	+		s
Ornithine carbamoyltransferase (*argF*)		s					+	s
Inducible ornithine decarboxylase (*ocdI*)							+	
Inducible ornithine decarboxylase (*speF*)	+							

Putrescine-ornithine antiporter *(potE)*							+	
Glutamate decarboxylase (*gadA*)		s						
Glutamate decarboxylase (*gadB*)		s	+	s	+		+	
Glutamate/ gamma-aminobutyrate antiporter (*gadC*)		s	s	s			+	
Tryptophan decarboxylase (RUMGNA_01526)							+	
Aminopeptidase C (*pepC*)	+	s	+	+	+	+	+	+
Aminopeptidase E (*pepE*)	+	s					+	+
Aminopeptidase N (*pepN*)	+	+	+	+	+	+	+	
Aminopeptidase S (*pepS)*	+							+
Aminopeptidase YpdF (*ypdF)*	+	s	+	s	+	+	+	+
Methionine aminopeptidase (*map*)	+	+	+		+	+	+	+
Carboxypeptidase (*ypwA*)						+		+
Neutral endopeptidase (*pepO*)	+	+	+	+	+	+	+	+
Peptidase T (*pepT*)	+	s	+	s	+	+	+	+
Glutamyl aminopeptidase (*pepA*)			+	+	+	+		+
Glutamyl endopeptidase (*gseA*)							+	

Supplementary Figures

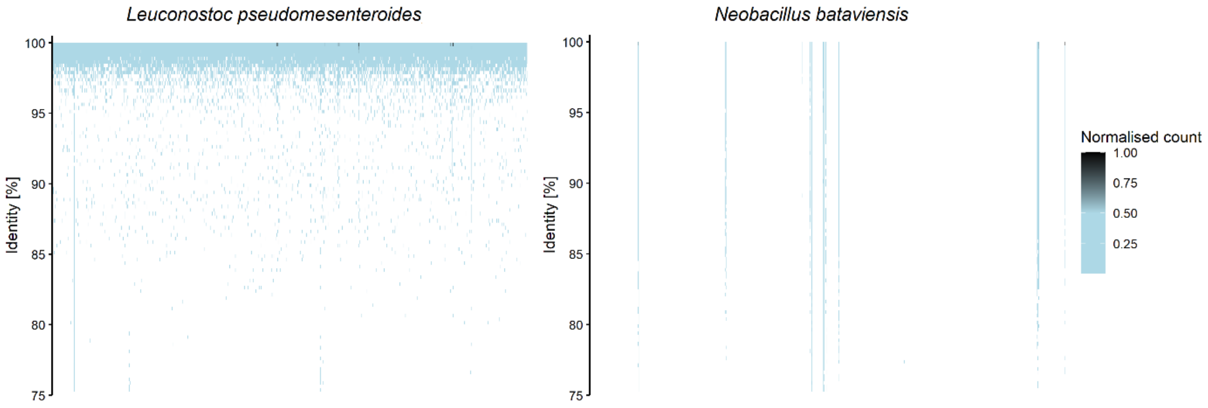

Figure S1. Example of metagenomic recruitment plots for two bacterial species of Gouda cheese sample A6 after 36 weeks of ripening (Gouda cheeses with batch-to-batch variability), obtained through short-read metagenomic sequencing. Each dot represents a bin of high-quality metagenomic sequence reads (HQ-MSRs) recruited by the reference genome of *Leuconostoc pseudomesenteroides* (4882) and *Neobacillus bataviensis* (LMG 21833), respectively. The X-axis represents the length of the reference genome. The Y-axis represents the percentage of sequence identity between the HQ-MSRs and the reference sequences. The color represents the number of reads per bin, normalized to the number of reads in the largest bin. As for *Leuc. pseudomesenteroides*, the whole genome length was covered at very high sequence identity values, this species was considered as present, whereas *N. bataviensis* was not considered as present, since only few positions in the whole genome were covered at varying sequence identity values.

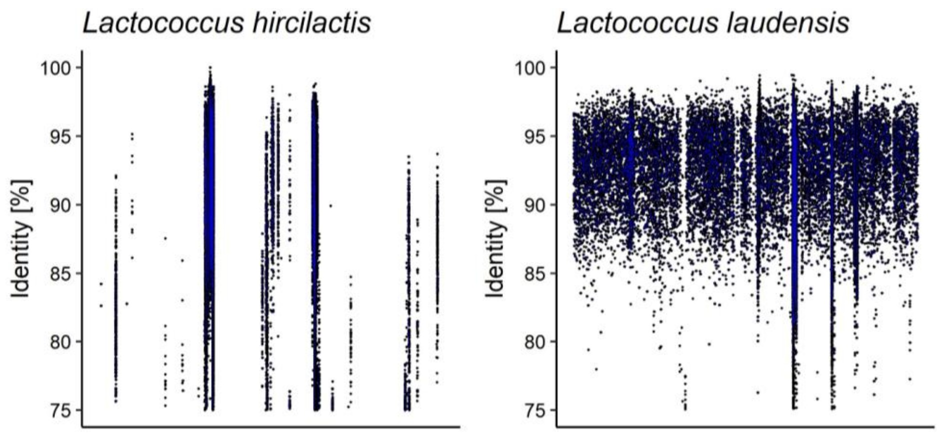

Figure S2. Example of metagenomic recruitment plots for two bacterial species present in the core of a cheese sample obtained after 45 weeks of ripening in the longitudinal study of a Gouda cheese production batch, based on long-read sequencing data. Each read recruited by the reference genomes is represented by a black dot at its start and end, connected by a blue line. The X-axis represents the length of the reference genomes, namely *Lactococcus hircilactis* DSM 28960 and *Lactococcus laudensis* DSM 28961. The Y-axis represents the percentage of sequence identity between the reads and the reference sequences. As for *Lc. laudensis*, the whole genome length was covered at very high sequence identity values, this species was considered as present, whereas *Lc. hircilactis* was not considered as present, since only few positions in the whole genome were covered at varying sequence identity values.

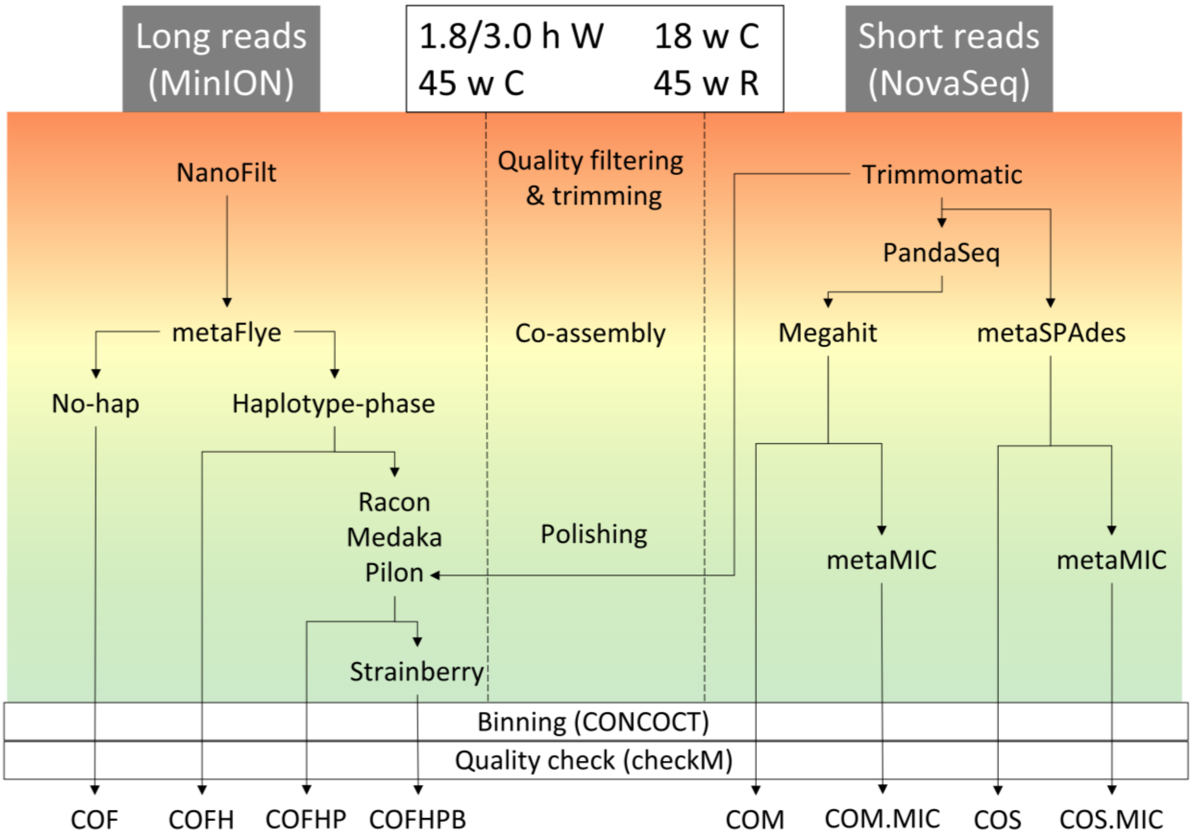

Figure S3. Overview of the metagenomic strategies applied to obtain high-quality metagenomic bins with long-read sequencing and short-read sequencing. COF, long-read co-assembly using metaFlye without considering haplotypes; COFH, long-read co-assembly using metaFlye considering haplotypes; COFHP, COFH assembly polished with short reads by means of Pilon; COFHPB, COFHP assembly processed by Strainberry; COM, short-read co-assembly using Megahit; COM.MIC, short-read co-assembly using Megahit curated with metaMIC; COS, short-read co-assembly using metaSPAdes; and COS.MIC, short-read co-assembly using metaSPAdes curated with metaMIC.

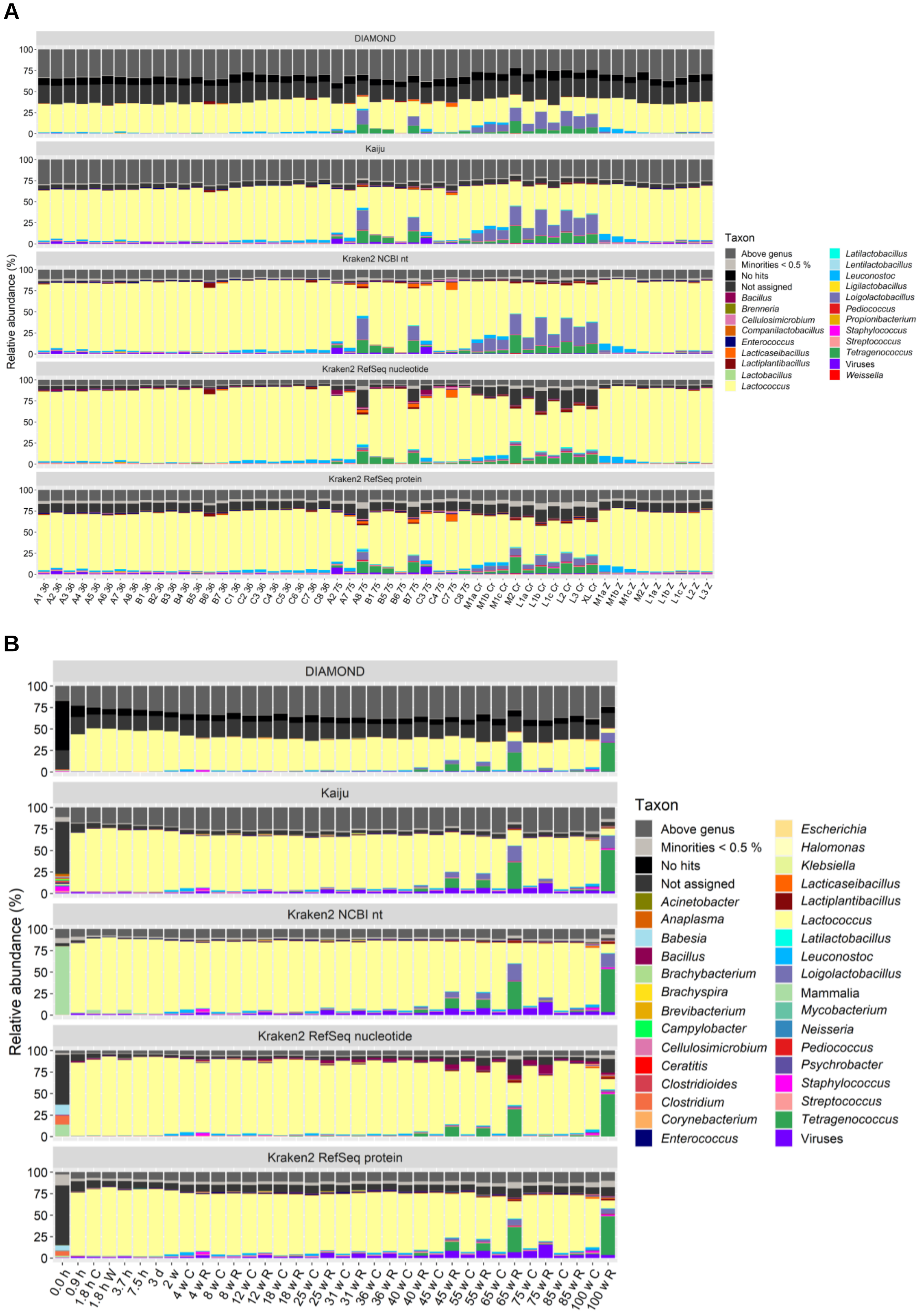

A
DIAMOND
Kaiju
Kraken2 NCBI nt
Kraken2 RefSeq nucleotide
Kraken2 RefSeq protein
Relative abundance (%)
0
25
50
75
100
A1 36
A2 36
A3 36
A4 36
A5 36
A6 36
A7 36
A8 36
B1 36
B2 36
B3 36
B4 36
B5 36
B6 36
B7 36
C1 36
C2 36
C3 36
C4 36
C5 36
C6 36
C7 36
C8 36
A2 75
A7 75
A8 75
B1 75
B5 75
B6 75
B7 75
C3 75
C4 75
C7 75
C8 75
M1a Cr
M1b Cr
M1c Cr
M2 Cr
L1a Cr
L1b Cr
L1c Cr
L2 Cr
L3 Cr
XL Cr
M1a Z
M1b Z
M1c Z
M2 Z
L1a Z
L1b Z
L1c Z
L2 Z
L3 Z
Taxon
Above genus
Minorities < 0.5 %
No hits
Not assigned
Bacillus
Brenneria
Cellulosimicrobium
Companilactobacillus
Enterococcus
Lacticaseibacillus
Lactiplantibacillus
Lactobacillus
Lactococcus
Latilactobacillus
Lentilactobacillus
Leuconostoc
Ligilactobacillus
Loigolactobacillus
Pediococcus
Propionibacterium
Staphylococcus
Streptococcus
Tetragenococcus
Viruses
Weissella
B
DIAMOND
Kaiju
Kraken2 NCBI nt
Kraken2 RefSeq nucleotide
Kraken2 RefSeq protein
Relative abundance (%)
0
25
50
75
100
0.0 h
0.9 h
1.8 h C
1.8 h W
3.7 h
7.5 h
3 d
2 w
4 w C
4 w R
8 w C
8 w R
12 w C
12 w R
18 w C
18 w R
25 w C
25 w R
31 w C
31 w R
36 w C
36 w R
40 w C
40 w R
45 w C
45 w R
55 w C
55 w R
65 w C
65 w R
75 w C
75 w R
85 w C
85 w R
100 w C
100 w R
Taxon
Above genus
Minorities < 0.5 %
No hits
Not assigned
Acinetobacter
Anaplasma
Babesia
Bacillus
Brachybacterium
Brachyspira
Brevibacterium
Campylobacter
Cellulosimicrobium
Ceratitis
Clostridioides
Clostridium
Corynebacterium
Enterococcus
Escherichia
Halomonas
Klebsiella
Lacticaseibacillus
Lactiplantibacillus
Lactococcus
Latilactobacillus
Leuconostoc
Loigolactobacillus
Mammalia
Mycobacterium
Neisseria
Pediococcus
Psychrobacter
Staphylococcus
Streptococcus
Tetragenococcus
Viruses

Figure S4. Taxonomic classification at the genus level of the high-quality metagenomic sequence reads for (**A**) the 23 Gouda cheeses with batch-to-batch variability and the cheeses with crack defects (names as explained in Table 1) and (**B**) the processed milk and cheese samples from the longitudinal study of a Gouda cheese production batch (C, curd/core; W, whey; R, rind), using DIAMOND, Kaiju, and Kraken2 [NCBI nucleotide database (NCBI nt), RefSeq nucleotide database (RefSeq nucleotide), or RefSeq protein database (RefSeq protein)]. The category "Minorities" includes all genera that were represented by less than 0.5 % of all reads for all methods applied. The category "Above genus" represents all taxonomic levels assigned above genus level. The category "No hits" includes all reads that could not be classified at all. The category "Not assigned" represents reads that could not be assigned to any taxonomic level.

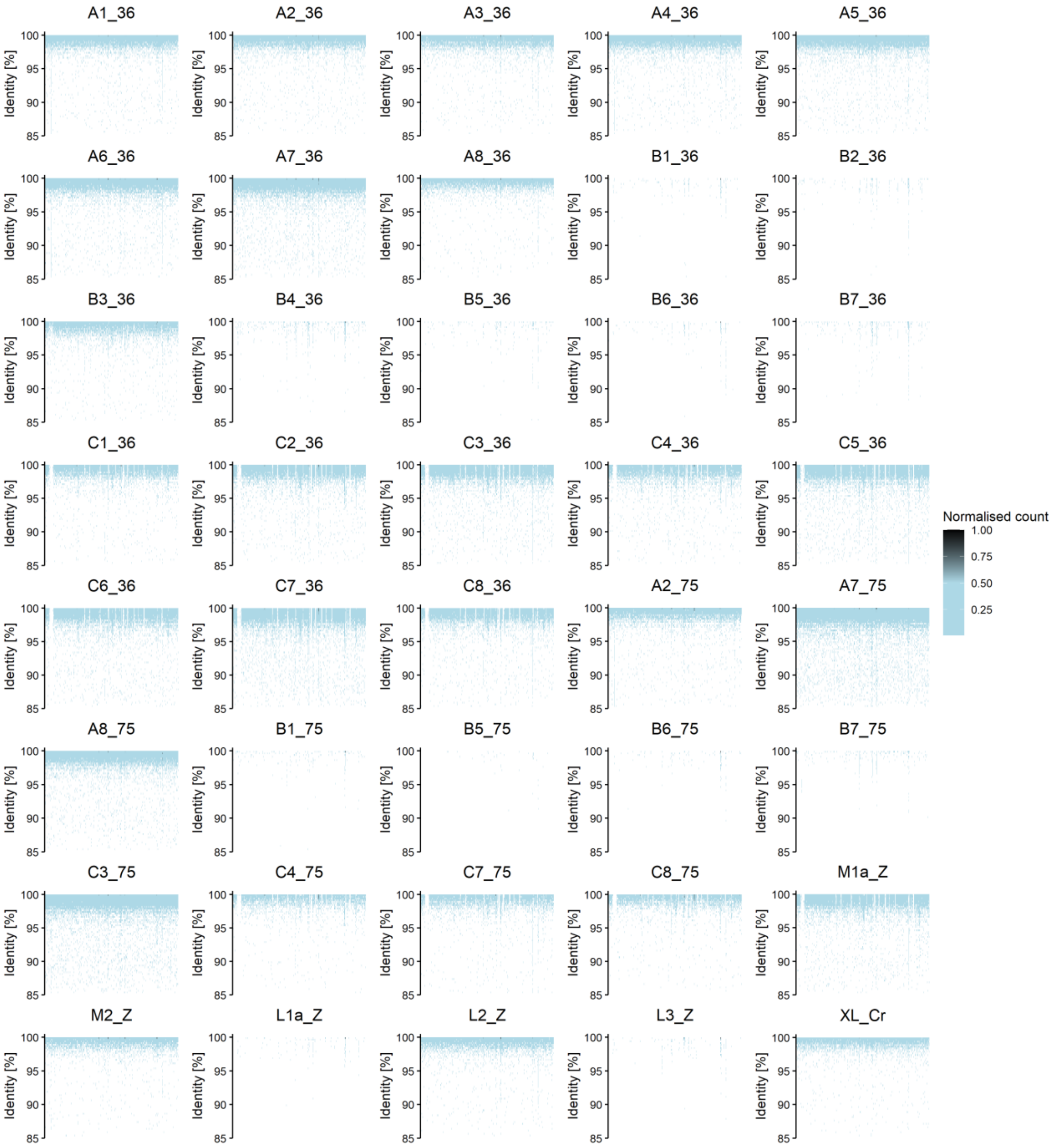

Figure S5. Metagenomic recruitment plots for the *Leuconostoc pseudomesenteroides* 4882 genome based on high-quality metagenomic sequence reads (HQ-MSRs) of 23 Gouda cheeses with batch-to-batch variability and cheeses with crack defects. The sample codes are as explained in Table 1. The X-axis represents the length of the reference genome. The Y-axis represents the sequence identity values between the HQ-MSRs and the reference genome. The color represents the number of reads per bin, normalized to the number of reads in the largest bin. Only one sample per batch is shown for the cheeses with crack defects (the plots of Gouda cheese wheel a, b, and c of M1 and L1 were similar, and the plots of the zones with cracks and the zones without cracks were similar as well).

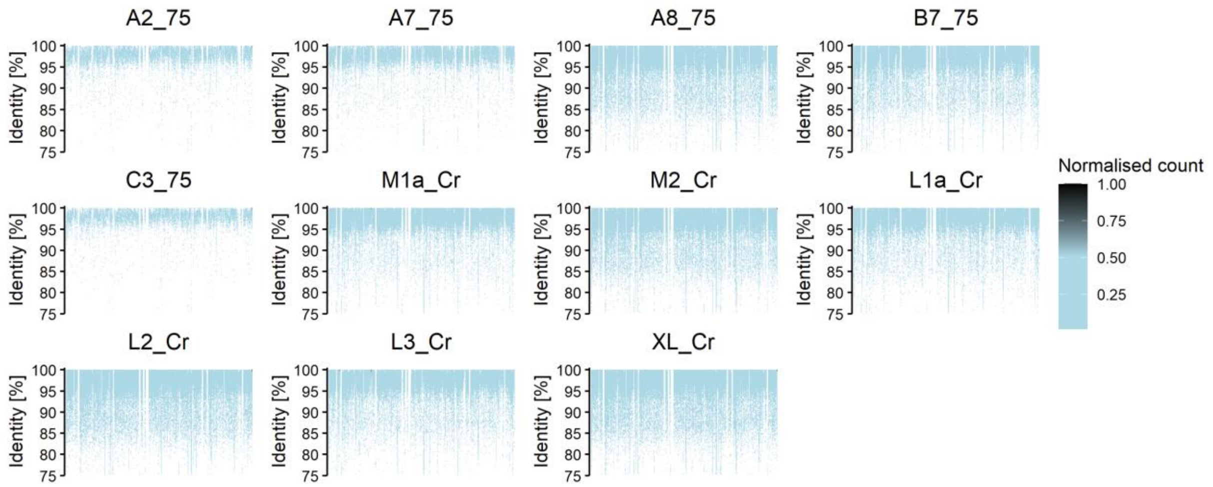

Figure S6. Metagenomic recruitment plots for the *Loigolactobacillus rennini* DSM 20253 genome based on high-quality metagenomic sequence reads (HQ-MSRs) of some of the 23 cheeses with batch-to-batch variability and cheeses with crack defects. Sample codes, axes, and colors are as explained in the legend of Figure S5.

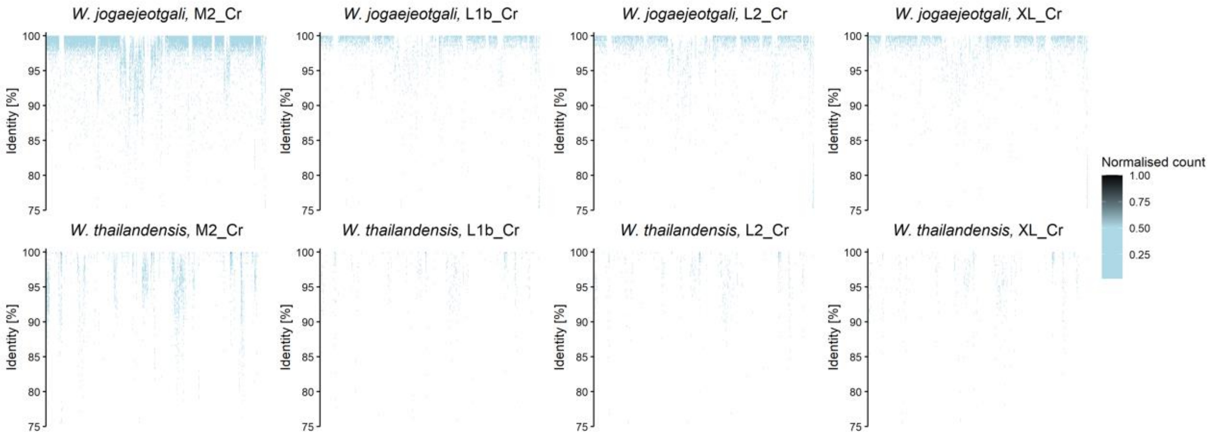

Figure S7. Metagenomic recruitment plots for the *Weissella jogaejeotgali* FOL01 and the *Weissella thailandensis* KCTC 3751 genomes based on high-quality metagenomic sequence reads (HQ-MSRs) of some of the cheeses with crack defects. Sample codes, axes, and colors are as explained in the legend of Figure S5.

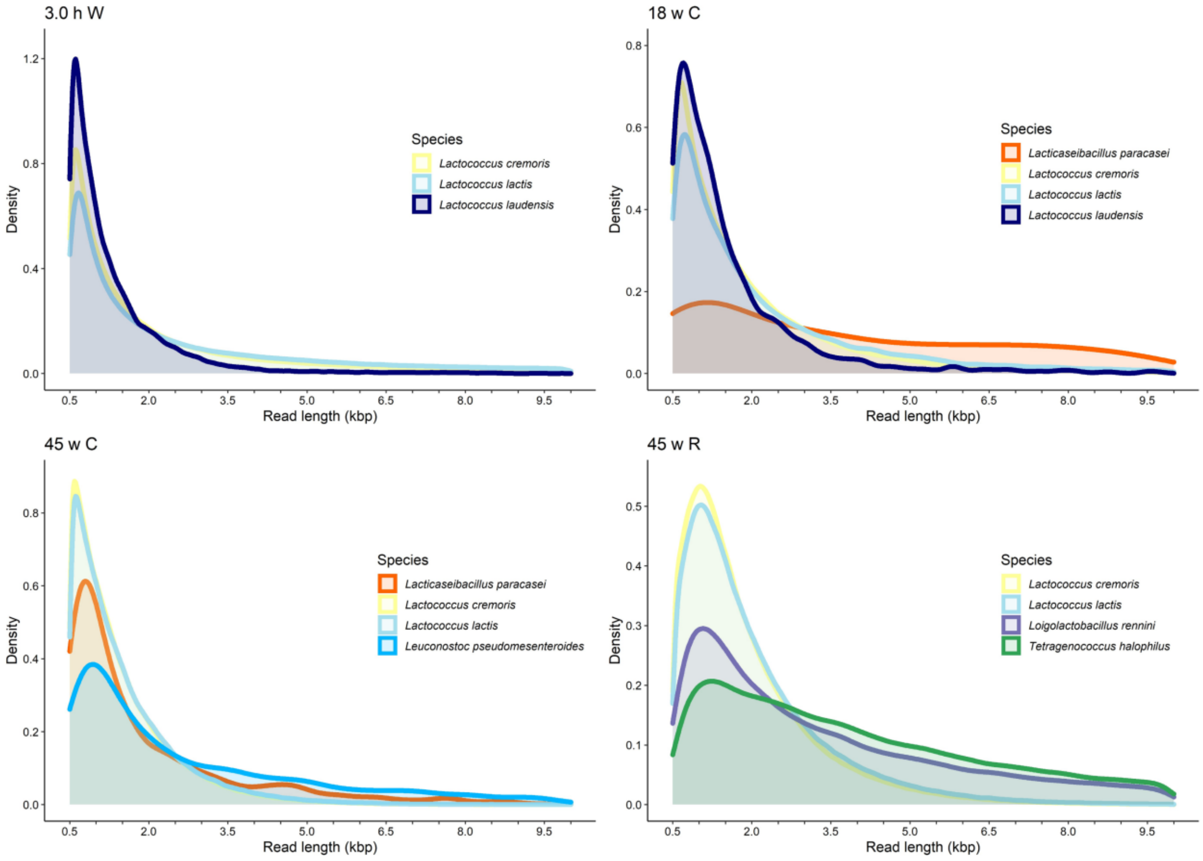

Figure S8. Density plots for the main species present in four whole-community DNA samples from the longitudinal study of a Gouda cheese production batch that were sequenced by long-read sequencing. The density represents the frequency of reads for each read length, per species. W, whey; C, core; R, rind.

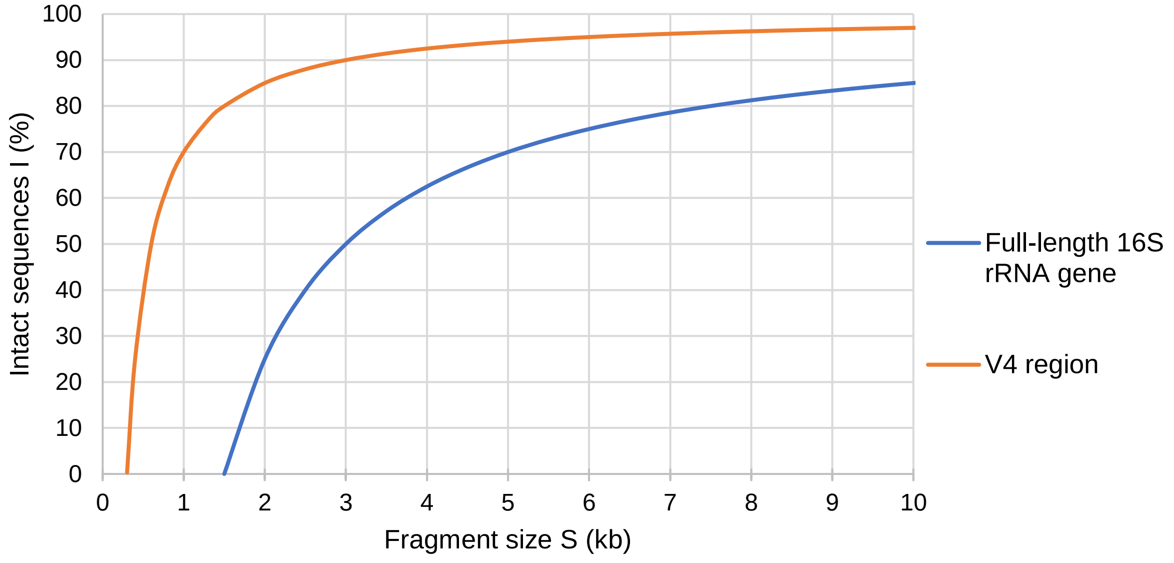

Figure S9. Simulation of the intact sequences *I* of the V4 region of the 16S rRNA gene, and the full-length 16S rRNA gene, respectively, in the hypothetical situation that all bacterial DNA is fragmented in equally long parts with fragment size *S*.

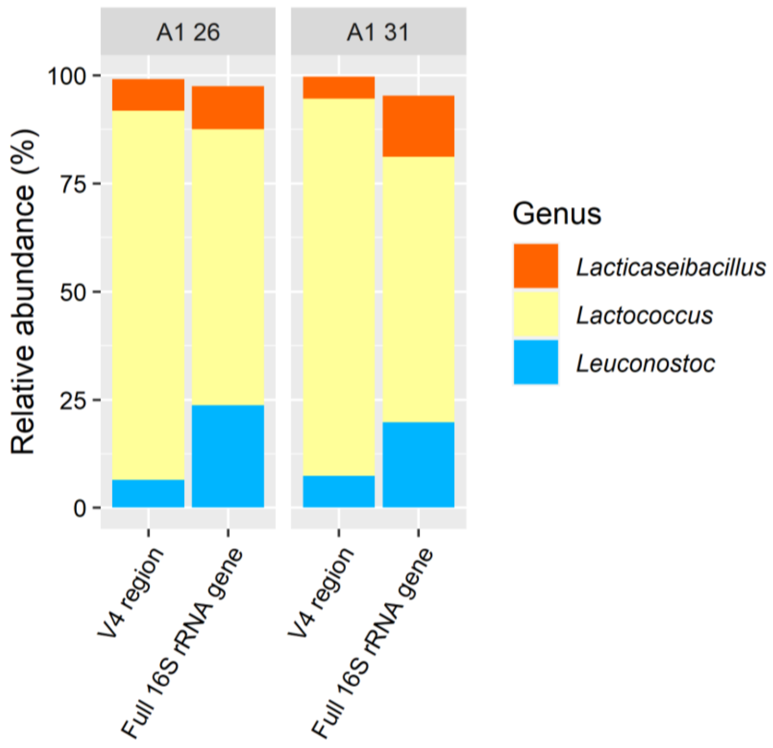

Figure S10. Genus-level identification of cheese sample A1 obtained after 26 and 31 weeks of ripening, according to high-throughput amplicon sequencing of the V4 region of the 16S rRNA gene, and the full-length 16S rRNA gene, respectively. Data from Decadt et al., 2023.

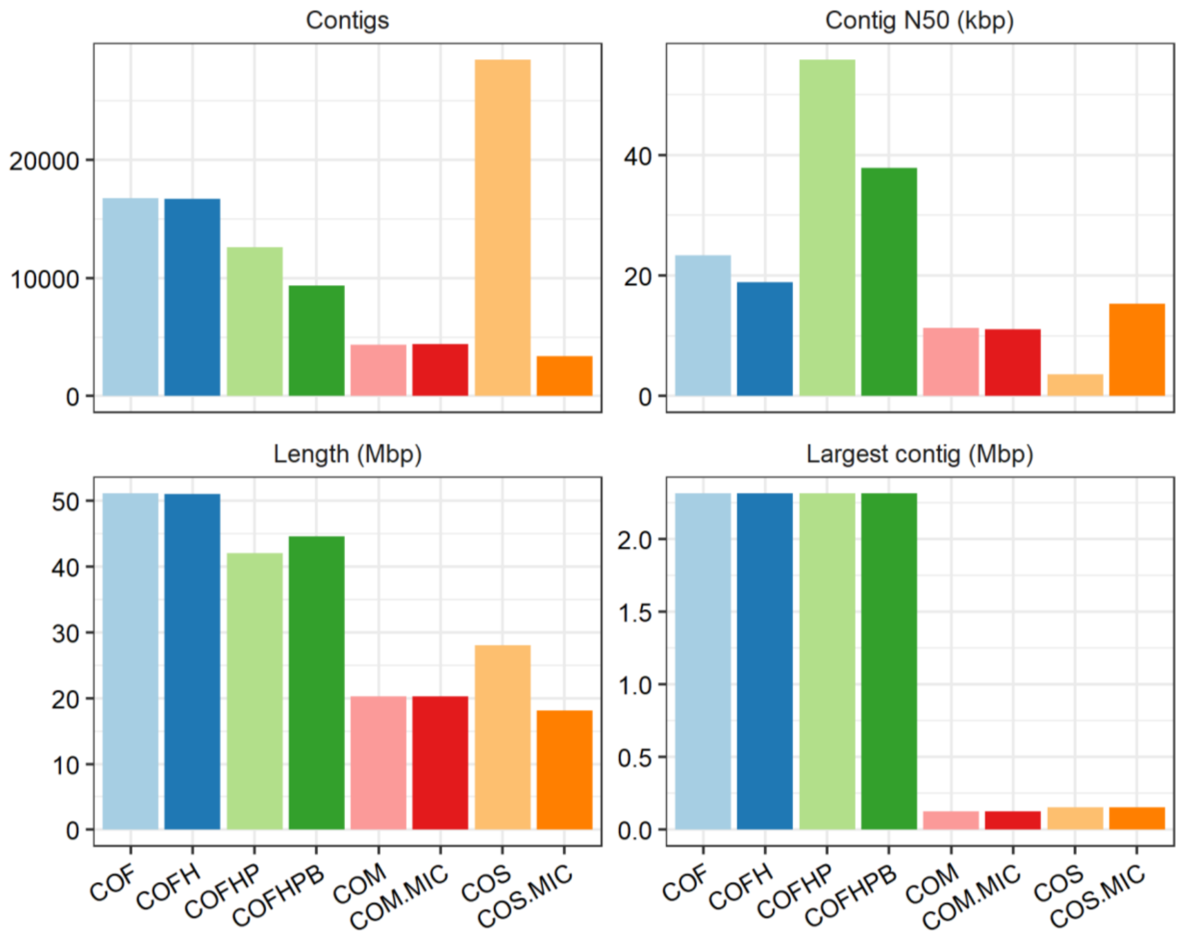

Figure S11. The main assembly statistics for the eight co-assemblies generated with long reads only, long reads polished with short reads, and short reads only for the four whole-community DNA cheese samples from the longitudinal study of a Gouda cheese production batch sequenced by both short- and long-read metagenomic sequencing. The co-assembly codes are as explained in the legend of Figure S3.

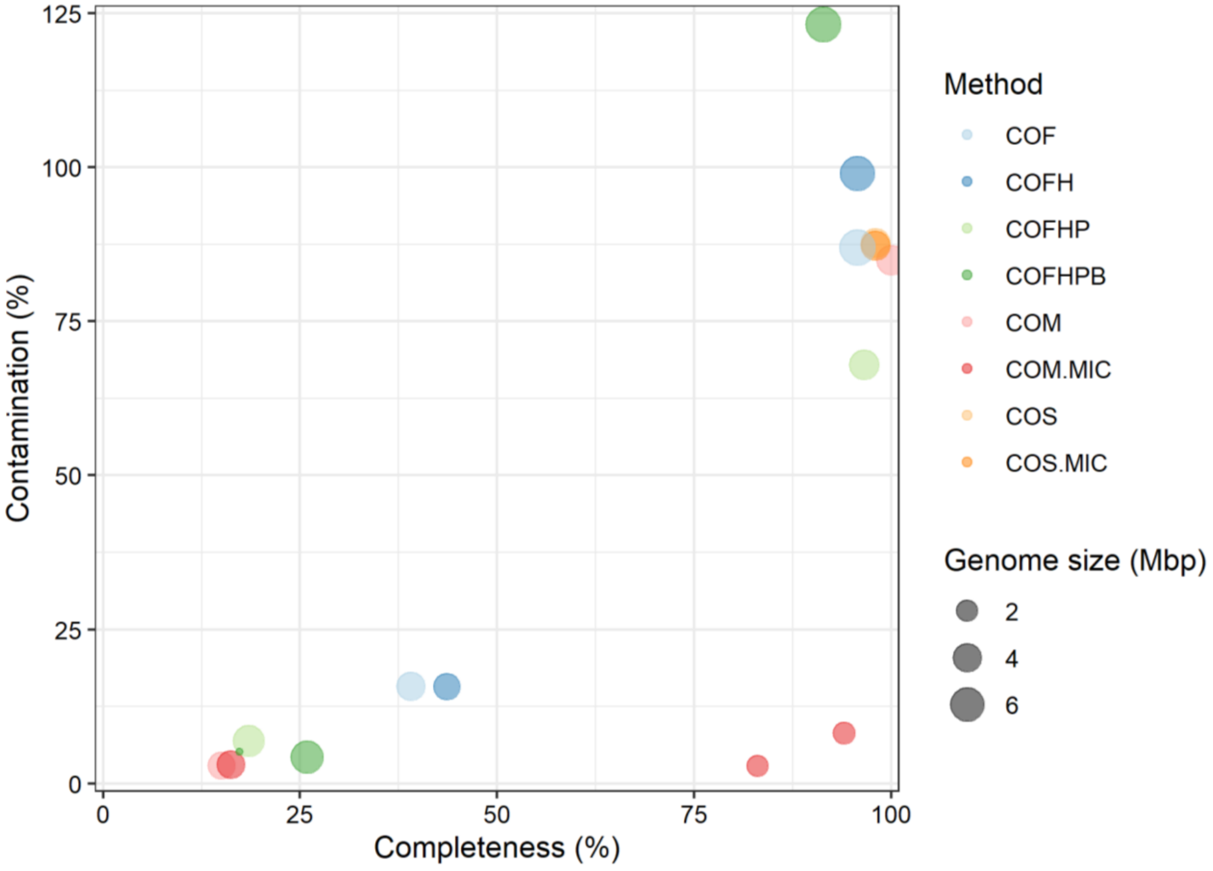

Figure S12. Percentages of completeness (X-axis) and contamination (redundancy) (Y-axis) of every metagenomic bin identified as *Lactococcus cremoris* by the eight co-assemblies generated with long reads only, a combination of long and short reads, and short reads only for the four whole-community DNA cheese samples from the longitudinal study of a Gouda cheese production batch sequenced by both short- and long-read metagenomic sequencing. The co-assembly code is as explained in the legend of Figure S3.

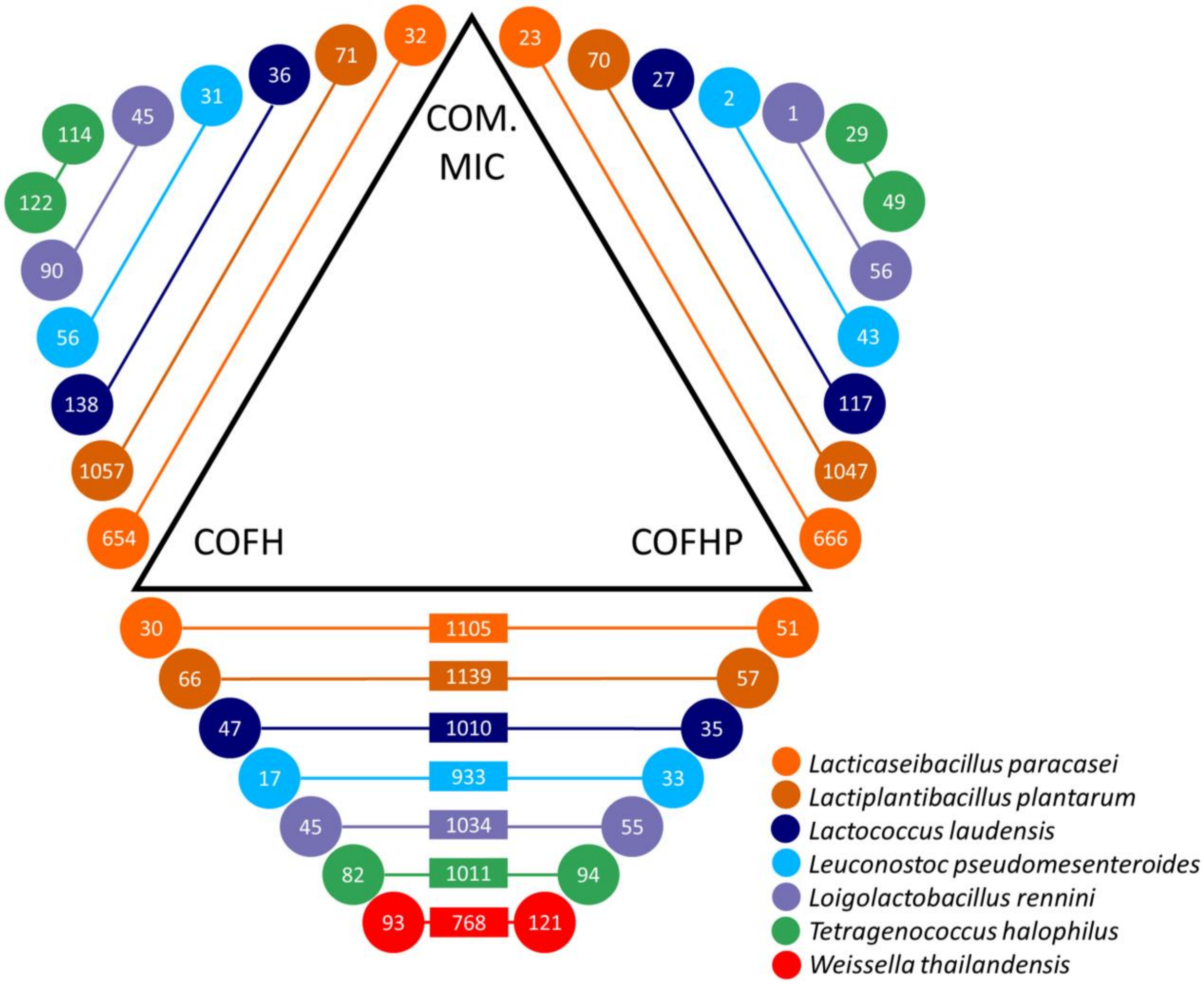

Figure S13. Pairwise comparison of the gene functions identified for each species by three different co-assembly methods, namely, COM.MIC (considered as the best short-read method), COFH (considered as the best long-read method), and COFHP (considered as the best method combining long and short reads), for the four whole-community DNA cheese samples from the longitudinal study of a Gouda cheese production batch sequenced by both short- and long-read metagenomic sequencing. The number of unique gene functions for each method is indicated in the corners, the number of shared gene functions between COFH and COFHP is indicated on the base (which can also be used to calculate the number of shared gene functions between the other pairs). Gene functions were predicted by Prokka for each species, based on the MAGs or metagenomic bins obtained by COM.MIC (short reads only), COFH (long reads only), and COFHP (long reads polished using short reads). Only defined gene functions were taken into account, hence excluding hypothetical proteins.
